# COVID-19: An Overview of SARS-CoV-2 Variants—The Current Vaccines and Drug Development

**DOI:** 10.1155/2023/1879554

**Published:** 2023-08-29

**Authors:** Narjess Bostanghadiri, Pardis Ziaeefar, Morvarid Golrokh Mofrad, Parsa Yousefzadeh, Ali Hashemi, Davood Darban-Sarokhalil

**Affiliations:** ^1^Department of Microbiology, School of Medicine, Iran University of Medical Sciences, Tehran, Iran; ^2^School of Medicine, Shahid Beheshti University of Medical Sciences, Tehran, Iran; ^3^Razi Vaccine and Serum Research Institute, Agricultural Research, Education and Extension Organization (AREEO), Karaj, Iran; ^4^Department of Microbiology, School of Medicine, Shahid Beheshti University of Medical Sciences, Tehran, Iran

## Abstract

The world is presently in crisis facing an outbreak of a health-threatening microorganism known as COVID-19, responsible for causing uncommon viral pneumonia in humans. The virus was first reported in Wuhan, China, in early December 2019, and it quickly became a global concern due to the pandemic. Challenges in this regard have been compounded by the emergence of several variants such as B.1.1.7, B.1.351, P1, and B.1.617, which show an increase in transmission power and resistance to therapies and vaccines. Ongoing researches are focused on developing and manufacturing standard treatment strategies and effective vaccines to control the pandemic. Despite developing several vaccines such as Pfizer/BioNTech and Moderna approved by the U.S. Food and Drug Administration (FDA) and other vaccines in phase 4 clinical trials, preventive measures are mandatory to control the COVID-19 pandemic. In this review, based on the latest findings, we will discuss different types of drugs as therapeutic options and confirmed or developing vaccine candidates against SARS-CoV-2. We also discuss in detail the challenges posed by the variants and their effect on therapeutic and preventive interventions.

## 1. Introduction

This new virus is called coronavirus due to its crown-like surface glycoprotein [1]. The coronavirus belongs to the Coronaviridae family and the Nidovirales order. It is a single-stranded, positive-sense RNA virus with four structural proteins, including envelope (E protein), membrane (M protein), nucleocapsid (N protein), and spike (S protein) [2]. These viruses primarily affect birds and mammals; they also cause the common cold among humans. Three human coronaviruses have emerged in the last twenty years, including SARS-COV and MERS-COV, leading to life-threatening pandemic situations [3]. COVID-19, which is caused by the novel severe acute respiratory syndrome coronavirus 2 (SARS-CoV-2), first originated in Wuhan, China, in December 2019; on 11 March 2020, the World Health Organization (WHO) declared a pandemic situation [4, 5]. The incubation period for SARS-CoV-2 ranges from 2 to 7 days [6]. About 80% of COVID-19 patients present with mild to moderate symptoms, while 20% of them manifest severe and critical symptoms [7].

For COVID-19 diagnosis confirmation, several laboratory tests, including nucleic acid amplification test (NAAT) such as rRT-PCR and rapid diagnostic tests based on antigen detection, such as lateral flow immunoassays (LFI) serological antibody assays, are accomplished on throat swabs, sputum, blood, stool, and other samples. For instance, upper respiratory specimens are used in the early stages of the disease. In contrast, lower respiratory specimens are used in more advanced stages of the disease or in patients who are highly suspicious of COVID-19 with a negative upper respiratory test [8]. Here, we review the pathogenesis and clinical manifestations of COVID-19, possible treatments, and confirmed or developing vaccine candidates.

## 2. Pathogenesis of SARS-CoV-2

The viral infection starts with the interaction of S protein and ACE2 on the host cell surface. Priming cleavage (between S1 and S2) and activating cleavage (on S2's site) by host proteases including furin, transmembrane protease serine protease-2 (TMPRSS-2), TMPRSS-4, cathepsins, trypsin, or human airway trypsin-like protease lead to the activation of viral S protein [9, 10]. After the fusion, the viral particles enter the cell by the endosomal pathway; the viral genome is released in the cytoplasm by a proteolytic cleft in the viral envelope. Subsequently, RNA is translated by host machinery to produce replicase and structural proteins. During infection, RdRp, 3CLpr, and PLpro are encoded by the viral genome. RdRp mediates SARS-CoV-2 RNA replication and amplification and translates viral polypeptides (pp1a and 1ab) and NSPs from the viral genome. PLpro suppresses the immune system by ubiquitinating specific host cell proteins, including interferon factor 3 and nuclear factor kappa B (NF-*κ*B) [11]. The viral genome is also translated into structural proteins (S, M, and N) to assemble a new virus. The translation and accumulation of M, E, and S proteins occur in the endoplasmic reticulum (ER) and ER-Golgi compartment (ERGIC). Eventually, the mature virion is released from the host cell via exocytosis [10, 12].

## 3. Clinical Manifestation

Clinical manifestation of COVID-19 is ranged from asymptomatic and mild to severe symptoms and mortality. Manifestations of COVID-19 are classified into two groups: respiratory and nonrespiratory symptoms. Pulmonary manifestations include dry cough, sputum cough, dyspnea, pneumonia, acute respiratory distress syndrome, and respiratory failure, and some systemic symptoms include fever, fatigue, weakness, myalgia, anosmia, and ague [13, 14]. Gastrointestinal (GI) manifestations include anorexia, nausea, vomiting, diarrhea, abdominal pain and discomfort, and gastrointestinal bleeding [15, 16]. Liver impairment which is described by elevation in aminotransferase, bilirubin, gamma-glutamyltransferase, hypoproteinemia, and prolonged prothrombin time are hepatic manifestation of COVID-19 [17, 18]. Also in a study carried out by Wang et al., pancreatic injury (abnormal lipase or amylase) due to COVID-19 infection occurred in 17% of patients [19]. Dermatological manifestations have been reported rarely including erythematous rash, urticarial, livedo reticularis, chicken-pox-like vesicles, pernio-like lesions, and petechiae, which mostly occur on the trunk [20–24] and also gangrene and finger or toe cyanosis, which occur in seriously ill patients [25]. Renal manifestations associated with COVID-19 include proteinuria, hematuria, hyponatremia, hypernatremia, hyperkalemia, and metabolic acidosis [26, 27]. A study conducted by Yang et al. indicated that acute kidney injury (AKI) occurs more likely among COVID-19 patients hospitalized in the intensive care unit (ICU) [13]. Moreover, Cardiac manifestations of COVID-19 include acute myocardial injury, myocarditis, arrhythmias (such as bradyarrhythmias, tachyarrhythmias, atrial fibrillation, and nonsustained ventricular tachycardias), prolonged QTc, Takotsubo Syndrome (TTS), heart failure, coronary heart disease, and cardiac arrest [28–31]. Neurological manifestations are classified into two groups: the central nervous system and the peripheral nervous system. Peripheral nervous system manifestations include neuralgia, hypogeusia, hyposmia, polyneuropathy, and Guillain-Barre syndrome [32–34]. In a study carried out by Frye et al., 5 patients with Guillain-Barre syndrome were reported whose neurological syndrome started within 5 to 10 days after the onset of the flu-like syndrome [35]. Central nervous manifestations include dizziness, headache, seizure, ataxia, transverse myelitis, encephalitis, and complications such as acute cerebrovascular disease, acute hemorrhagic necrotizing encephalopathy, and impaired consciousness [36–39]. Manifestations of ocular due to COVID-19 have rarely been reported in case reports and series which indicate symptoms of acute conjunctivitis such as hyperemia, chemosis, epiphora, and foreign body sensation [40, 41].

## 4. SARS-CoV-2 Variants

Similar to other RNA viruses, SARS-CoV-2, while being competent in human cells, can modify their genomic sequence while undergoing replication within host cells. These modifications, referred to as mutations, may exhibit distinct traits when compared to their ancestral counterparts [42]. The genetic evolution of variants initially started with a single D614G mutation and a 69/70 deletion in the S glycoprotein in China and Denmark, respectively [43–45]. The emergence of new SARS-CoV-2 variants is a worrying issue due to the numerous mutations that occur throughout the viral genome. The variants have been named in several ways based on different nomenclature systems. Three naming systems for SARS-CoV-2 variants are currently in use: Pango [46], the global initiative on sharing all influenza data (GISAID) [47], and Nextstrain [48]. The prevailing system utilized for categorizing lineages is Pango. The WHO, in collaboration with the SARS-CoV-2 Interagency Group (SIG) and the Centers for Disease Control and Prevention (CDC), devised a classification system for SARS-CoV-2 variants. This system categorizes variants as “Variants of Interest (VOI),” “Variants of Concern (VOC),” “Variants of High Consequence (VOHC),” and “Variants Being Monitored (VBM)” [49]. Additionally, in May 2021, the WHO recommended the use of Greek letters in conjunction with the scientific names for VOI and VOC [50]. On the 15th of March 2023, the WHO made an official declaration stating that, henceforth, the Greek alphabet will solely be utilized to assign designations to VOCs, while VOIs will be denoted by means of established scientific nomenclature systems, such as those employed by Nextstrain and Pango [51].

### 4.1. Variants Being Monitored

Variants that possess data indicating a potential or evident influence on approved or authorized medical countermeasures or have been correlated with more severe illness or heightened transmission, yet are presently undetectable or circulating at exceedingly low levels in the United States, do not pose a significant or imminent hazard to public health in the United States [49].

### 4.2. Variants of Interest

A variant possessing particular genetic markers that have been linked with alterations to receptor binding, decreased neutralization by antibodies generated in response to previous infection or immunization, reduced therapeutic effectiveness, potential diagnostic consequences, or an anticipated escalation in transmissibility or disease severity. The VOI may possess the following attributes: specific genetic markers that are expected to influence immune escape, diagnostics, therapeutics, or transmission; evidence indicating that it is accountable for an amplified proportion of cases or distinctive outbreak clusters; and limited occurrence or expansion in the United States or other nations [49, 52] (Table 1).

### 4.3. Variants of High Consequence

A variant of notable significance has presented clear evidence indicating that measures for prevention or medical countermeasures (MCMs) have experienced a significant decline in effectiveness when compared to previously circulating variants. This particular variant may possess additional attributes, such as the demonstrated inadequacy of diagnostic methods, evidence suggesting a noteworthy reduction in vaccine effectiveness, an overabundance of vaccine breakthrough cases, or an insufficient vaccine-induced safeguard against severe disease. Additionally, this variant may exhibit a significantly reduced susceptibility to various EUA or approved therapeutics, leading to more severe clinical disease and an increase in hospitalizations [49, 53].

### 4.4. Variants of Concern

A variant that exhibits indications of heightened transmissibility, exacerbated illness (including increased hospitalizations or fatalities), a significant reduction in neutralization by antibodies produced during prior infection or vaccination, lowered efficacy of treatments or vaccines, or failures in diagnostic detection represents a VOC. Additional features of a VOC (in conjunction with those of a variant of interest) may include a demonstrable impact on diagnostics, treatments, and vaccines; pervasive interference with diagnostic test targets; evidence of markedly increased resistance to one or more categories of therapies; evidence of considerable decreased neutralization by antibodies produced during prior infection or vaccination; evidence of reduced vaccine-initiated protection against severe illness; indications of elevated transmissibility; and indications of heightened disease severity. According to the most current reports published by the World Health Organization at the time of composition, certain significant variants of the SARS-CoV-2 virus have been distinguished and will be examined within this particular section. [49, 52] (Table 2).

#### 4.4.1. Alpha (B.1.1.7)

On December 14, 2020, a new SARS-CoV-2 variant belonging to the B.1.1.7 lineage was first circulated in Kent and Greater London, the United Kingdom, and has been named in several ways based on different nomenclature systems [54] (Table 2). The B.1.1.7 lineage has been classified as a VOC associated with increased transmissibility and disease severity but with minimal impact on neutralization by convalescent and postvaccination sera [49, 55, 56].

**Figure 1:**
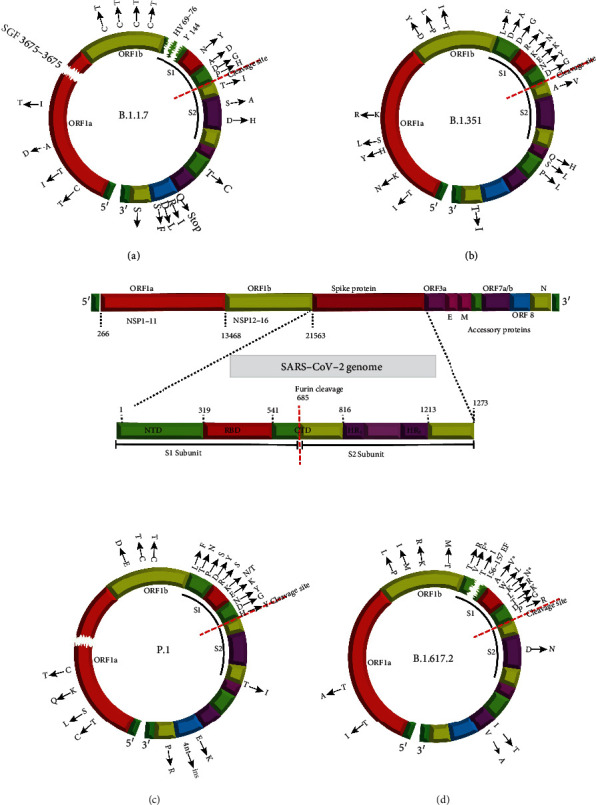
Organization of SARS-CoV-2 genome, components of spike (S) protein, and variants with identified mutation sites in the structural region.

As reported, this variant has acquired 17 mutations in its genome that lead to amino acid replacements and four deletions located in the ORF 1 a/b, ORF 8, spike protein, and M and N gene regions [57] (Figure 1(a)). Several of these mutations are in the S protein such as H69-V70, Y144 deletion (del), and N501Y, A570D, P681H, T716I, S982A, and D1118H amino acid substitutions. Key mutations in spike protein are N501Y, H69-V70 del, and P681H. The N501Y mutation in RBD increased the affinity of viral binding to the hACE2 receptor [58]. According to preliminary evidence, the N501Y mutation (a change from asparagine (N) to tyrosine (Y) in amino-acid position 501) may increase transmissibility [59, 60]. Gu et al. conducted that the N501Y mutation has also increased infectivity and virulence in a mouse model [61]. This mutation has also been observed in B.1.351 and P.1 variants. As we mentioned above, when S proteins are assembled on the surface of a coronavirus, they are not yet ready to attach to a cell. A human protease enzyme must first cut apart a section of the spike stem. The P681H mutation, which is adjacent to the protease cleavage site, facilitates the access of human proteases to the cleavage site, thereby increasing the transmission and infection of SARS-CoV-2 [62, 63]. On the other hand, it threatens the stability of the S protein [57]. One mysterious mutation in the B.1.1.7 lineage is H69/V70 deletions in the N-terminal domain (NTD), which seems to facilitate the escape of the coronavirus from the host's immunity response and diminish the susceptibility to NTD-specific neutralizing antibodies [64]. A combination of H69/V70 del and N501Y was shown to increase infectivity in vitro [63]. According to several studies, the deletion of Y144 has eliminated binding to neutralizing antibodies [64–66]. Therefore, the presence of these three deletions leads to a significant resistance of B.1.1.7 to NTD-specific neutralization antibodies. This VoC retains in vitro susceptibility to all the anti-SARS-CoV-2 monoclonal antibodies that are currently available through EUAs [67]. Ralimetinib, plitidepsin, and remdesivir possess antiviral efficacy against this variant [68].

#### 4.4.2. Beta (B.1.351)

The mutant strain B.1.351, also referred to as the beta variant or GH501Y.V2, was first isolated and identified in South Africa and reported by the country's health department [69]. This variant emerged with 21 mutations, of which nine mutations were located in the S protein region [70] (Figure 1(b)). Three mutations of particular interest in RBD (K417N, E484K, and N501Y), which help the virus latch on more tightly to human cells, and a further five spike mutations which have so far generated less concern include L18F, D80A, D215G, R246I, and A701V [69]. According to some evidence, immune evasion in the new B.1.351 strain, which has E484K and K417N mutations, occurs through increased in vitro resistance to some kinds of antibody neutralization such as bamlanivimab plus etesevimab and casirivimab, although sotrovimab and the combination of casirivimab and imdevimab appear to retain activity [71, 72]. Greaney et al. mentioned that the E484k change could significantly avoid recognition by polyclonal human serum antibodies [73]. Scientists are concerned about the variant because it is associated with an increased risk of transmission and a reduction in neutralization by monoclonal antibody therapy, convalescent serums, and postvaccination sera [42].

#### 4.4.3. Gamma (P.1)

A third variant of concern, the P.1 variant, is a branch of the B.1.1.28 lineage, also known as GR/501Y.V3 or the gamma variant, and was first detected in Brazil [74]. This strain carries 21 mutations, more than ten of which emerged in the spike protein, and the rest of them are located in the ORF 1 a/b, ORF 8, and N gene regions. Overall, three mutations are located in the RBD which are similar to the B.1.351 variant and designated as being of particular concern. The other changes across the spike include L18F, T20N, P26S, D138Y, R190S, H655Y, and T1027I (Figure 1(c)). As the P.1 variant shares three mutations in the RBD region, it significantly reduced susceptibility to antispike-neutralizing monoclonal antibodies, such as bamlanivimab alone and/or in combination with etesevimab, due to the E484K mutation. In vitro studies also suggest that the K417T and E484 mutations reduce casirivimab activity, although sotrovimab and the combination of casirivimab and imdevimab seem to preserve activity [75–77].

#### 4.4.4. Delta (B.1.617.2)

The fourth variant of concern, the SARS-CoV-2 delta variant, also known as lineage B.1.617.2, was first detected in Maharashtra, India, in late 2020 [78]. According to the CDC final update on 3 August 2021, B.1.617.2 harbors fifteen mutations in the S protein including T19R, (V70F^∗^), T95I, G142D, E156-, F157-, R158G, (A222V^∗^), (W258L^∗^), (K417N^∗^), L452R, T478K, D614G, P681R, and D950N, some of which were detected in some sequences but not all [49] (Figure 1(d)). Four of them are of particular concern such as D614G, T478K, L452R, and P681R. The L452R mutation confers a stronger affinity of the spike protein for the ACE2 receptor and decreased recognition capability of the immune system [45]. The results of studies show that L452R changes led to a modest decrease in in vitro susceptibility to the combination of bamlanivimab and etesevimab, although the clinical implications of this finding are not fully known. Sotrovimab and casirivimab plus imdevimab appear to retain activity [67, 79, 80]. The delta variant has been predicted to be 40-60% more transmissible than the alpha one. In mid-March 2021, a sublineage of the delta variant was detected in India and named the Delta plus (B.1.617.2.1) variant. It has acquired the K417N mutation which is also found in the beta variant [81].

## 5. Antiviral Therapy against COVID-19

**Figure 2:**
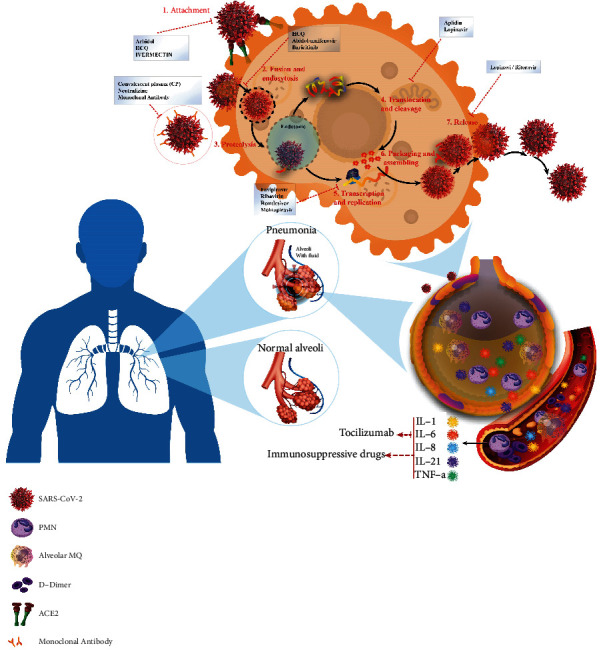
Replication of SARS-CoV-2 in target cells and the effect of drugs against COVID-19.

SARS-CoV-2 is a highly contagious agent with high mortality. To date, there are no clinically approved therapeutics in the U.S. Food and Drug Administration (FDA) specifically for COVID-19 [12, 82]. Only remdesivir was approved by the FDA for the treatment of this infection in hospitalized adults and hospitalized pediatric patients for at least 12 years. Remdesivir was originally invented for hepatitis C and subsequently for Ebola virus disease and Marburg virus. Therefore, it is not made specifically for the treatment of COVID-19. Developing a new drug with high efficacy and low toxicity from scratch to face the global pandemic challenge is impossible. Therefore, the drug repurposing method based on the similarity of disease mechanisms is an emerging strategy that provides a new treatment option for SARS-CoV-2 more rapidly than developing a new drug [83, 84]. As shown in Figure 2, preventing viral entry and fusion into host cells and suppressing the various steps of viral replication within cells are the two proposed target sites for repurposing conventional drugs and producing new therapeutic agents [85]. The mechanism, viral targets, and primary usage of the potential drugs are listed in Table 3.

### 5.1. Viral Entry and Fusion Inhibitors

#### 5.1.1. Chloroquine/Hydroxychloroquine (CQ/HCQ)

HCQ has been prescribed for long-term diseases including rheumatoid arthritis, lupus erythematous, and malaria [86, 87]. When SARS-CoV-2 emerged, attention was drawn to the antiviral aspects of HCQ/CQ, and scientists started to perform its efficacy for the treatment of COVID-19. Previously, the anti-inflammatory role of HCQ/CQ has been well established such as the inhibition of T and B cell receptors and toll-like receptor signals and the decrease of cytokine production such as IL-1, IL-6, and TNF-*α* [88]. It seems that HCQ has a role in the inhibition of ACE2 glycosylation, conversion of early endosome into late endosome, and formation of autophagosome against SARS-CoV-2 infection [89]. Despite reported benefits, a meta-analysis of randomized trials found that HCQ treatment is associated with accelerated mortality in COVID-19 patients and has no advantage over HCQ/CQ [90–92].

#### 5.1.2. Arbidol-Umifenovir

Umifenovir is an indole-based antiviral agent which interacts with its aromatic residues with the viral glycoproteins and inhibits viral entry and infectivity [93, 94]. Umifenovir (known as Arbidol) has a wide range of antiviral activity against influenza A, B, hepatitis B, C, Zika virus, Ebola virus, Lassa virus, poliovirus, and herpesvirus-8 [95–98]. Nojomi et al. performed an open-label randomized controlled trial of one hundred eligible patients with the diagnosis of COVID-19 in an educational hospital to examine the efficacy of ARB in patients with COVID-19. They divided patients into two groups and treated them with either hydroxychloroquine followed by Kaletra (lopinavir/ritonavir) or hydroxychloroquine followed by ARB. This study indicated better outcomes of ARB administration rather than Kaletra administration. These outcomes include clinical manifestation improvements, shorter hospital length of stay, less ICU admission indication, and more minor lung CT involvements [99]. A retrospective cohort study also found that timely intake of Arbidol in combination with interferon could reduce the days of virus shedding in patients with COVID-19 [100]. In another study, Arbidol had no significant effect on COVID-19 but showed a slight benefit in improving CT and side effects [101].

#### 5.1.3. Ivermectin

Ivermectin has previously been considered an in vitro inhibitor of some positive single-stranded RNA virus replication, including HIV, dengue, influenza, Zika virus, and yellow fever virus [102, 103]. But, originally, it is a broad-spectrum antiparasitic agent [104]. In March 2021, the latest update of WHO recommendations for COVID-19 treatments advised that ivermectin be used only in clinical trials. Because a guideline development group surveyed pooled data from 16 randomized controlled trials (total enrolled 2407) including both COVID-19 inpatients and outpatients, they found a very low certainty about the efficacy of ivermectin in reducing mortality, the need for mechanical ventilation, and the need for hospital admission [105, 106]. According to the last update of some selected clinical data, among outpatients with COVID-19, ivermectin did not shorten the time to sustained recovery, reduce incidence, or prevent the composite endpoint of hypoxemia, ED visit, hospitalization, or death [107–110].

Compared to CQ or HCQ, ivermectin did not reduce the proportion of hospitalized patients with severe COVID-19 who died or who required supplemental oxygen during ICU admission [111]. Unfortunately, according to the last update on March 6, 2023, ivermectin is not approved or authorized by the FDA for the treatment of COVID-19 [112].

### 5.2. Viral Protease Inhibitor

#### 5.2.1. Lopinavir/Ritonavir

Lopinavir/ritonavir (LPV/r), a well-known combination protease inhibitor, is the FDA-approved treatment for patients with HIV [113]. Studies have also reported its *in vitro* antiviral activity against SARS-CoV and MERS-CoV [114, 115]. They are classified as protease inhibitors which inhibit the SARS-CoV 3C-like protease enzyme [116]. Various studies have been performed to evaluate the efficacy and safety of lopinavir, as well as its simultaneous use with chloroquine, but the majority of studies have failed to provide evidence of their effectiveness [117, 118]. Some studies have suggested that it may be effective in reducing viral load and improving clinical outcomes, while others have found no significant benefit.

For example, a randomized controlled trial conducted in China found that lopinavir/ritonavir did not significantly improve clinical outcomes or reduce mortality compared to standard care alone. The study included 199 patients with severe COVID-19 who were randomly assigned to receive either lopinavir/ritonavir plus standard care or standard care alone. The researchers found no significant difference in the time to clinical improvement, mortality rate, or viral clearance between the two groups. Also, gastrointestinal adverse events were more common in the lopinavir-ritonavir group; however, serious adverse events occur mostly in the standard-care group [119]. Another study conducted research on treating patients with COVID-19 with a combination therapy of interferon beta-1b, lopinavir-ritonavir, and ribavirin. No serious adverse events were reported. Only self-limited nausea and diarrhea were seen which were not significant. e Another study which investigated the efficacy of lopinavir/ritonavir or Arbidol among 86 adult patients with mild/moderate COVID-19 indicated little benefit in improving clinical outcomes in patients consuming the aforementioned medications. Also, patients using lopinavir/ritonavir experienced some adverse events including elevation of ALT over 2.5-fold above the normal limit, diarrhea, and loss of appetite [121].

#### 5.2.2. Darunavir

Darunavir, a second-generation protease inhibitor drug often used in combination with cobicistat to enhance therapeutic effectiveness, is being studied as a potential treatment for SARS-CoV-2 due to promising in vitro results [122]. However, patients receiving darunavir/cobicistat have reported mild diarrhea and renal dysfunction compared to those receiving standard care [123]. Additionally, recent research has linked darunavir usage to an increased risk of myocardial infarction in HIV patients, suggesting that it may raise the risk of cardiovascular disease (CVD) [124]. A small randomized clinical trial in China involving mild COVID-19 patients treated with darunavir/cobicistat for five days on top of interferon-alpha 2b inhalation or interferon alpha 2b inhalation alone did not demonstrate any increase in the proportion of negative conversion compared to standard of care alone [123]. Also, an observational study in Qatar involving adult patients hospitalized for COVID-19 found that early treatment with lopinavir-ritonavir resulted in faster clinical improvement and/or virological clearance than darunavir/cobicistat [125]. Furthermore, a large cohort study of Italian patients hospitalized in 33 hospitals found that while the use of lopinavir/ritonavir did not change the death rate, the use of darunavir/cobicistat increased mortality [126].

#### 5.2.3. Nelfinavir

Nelfinavir, an HIV protease inhibitor, was evaluated for its effectiveness against SARS-CoV-2 during the COVID-19 pandemic. A study showed that the drug significantly reduced virus-induced cell-to-cell fusion in Vero cells infected with SARS-CoV-2 at a dose of 10 *μ*M, without affecting the cell surface expression of NMYC or FLAG glycoproteins. They noticed that administering nelfinavir early in the course of COVID-19 could limit virus spread and provide time for the immune system to respond [127]. In another in vitro study, it was found that combining nelfinavir with cepharanthine enhanced their predicted efficacy to control viral proliferation, potentially improving disease progression and reducing the risk of transmission [128].

#### 5.2.4. Atazanavir

Another protease inhibitor used to prevent and cure HIV is atazanavir. In a study by Beck et al., they found that atazanavir had the highest affinity for the 3CLpro of SARS-CoV-2 among a set of medications that also included remdesivir, efavirenz, ritonavir, and dolutegravir. They made this discovery using MT-DTI, a deep learning-based drug-target interaction prediction model. Atazanavir has also shown a potential affinity for practically all SARS-CoV-2 replication components in this model, including RdRp, helicase, exonuclease, and endoRNAse [129]. The effectiveness and safety of repurposed nitazoxanide in combination with atazanavir/ritonavir were assessed in a trial carried out in Nigeria among mild to moderate COVID-19 patients. In treating COVID-19, nitazoxanide coadministered with atazanavir/ritonavir was safe but ineffective compared to standard therapy [130]. In a study involving 62 patients with moderate-to-severe COVID-19, the patients were divided into two groups: KH (receiving lopinavir/ritonavir [Kaletra] plus hydroxychloroquine) and ADH (receiving atazanavir/ritonavir, dolutegravir, and hydroxychloroquine). According to the study's findings, the lopinavir/ritonavir therapy regimen may result in a less severe disease course than the atazanavir/dolutegravir treatment regimen, and it can be thought of as a complementary therapeutic option [131]. In a retrospective research, 170 individuals were divided into two therapy groups: group one received Kaletra plus HCQ, while group two received atazanavir/ritonavir (ATV) HCQ. Both groups show aberrant liver function in terms of safety. Also observed in group one were cases of nausea and diarrhea. Additionally, hospitalization and mortality between the two groups were not statistically significant in terms of efficacy. With regard to improved GI tolerance and fewer daily medications, the ATV regimen is preferable to Kaletra [132]. The efficacy and safety of hydroxychloroquine plus atazanavir/ritonavir in patients with moderate and severe COVID-19 were compared in a study among 213 participants. The use of hydroxychloroquine combined with atazanavir/ritonavir in patients with SpO2 below 90% at the time of hospital admission was not supported by this study. When patients were given hydroxychloroquine along with atazanavir/ritonavir, SpO2 was the only factor that could predict clinical outcomes, including length of hospital stay, discharge from the hospital, and mortality. The treatment protocol's gastrointestinal adverse effects (such as nausea, vomiting, abdominal pain, and diarrhea) were the most common [133]. In a control trial, 132 patients with moderate COVID-19 were divided into two groups and given either a single dose of HCQ plus atazanavir/ritonavir (ATZ/RTV) or lopinavir/ritonavir (LPV/RTV) for a minimum of five to a maximum of ten days. The rate and duration of ICU admission, intubation, recovery within 14 days, and mortality were outcomes that did not substantially differ between the two groups. ATZ/RTV were well tolerated, and the LPV/RTV group also experienced adverse effects (vomiting and nausea), although they were no more effective than LPV/RTV [134]. Regarding drug safety in patients administrating antiplatelet and new oral anticoagulants, atazanavir and LPV/r in COVID-19 patients should be used with caution [135]. In these patients receiving antiplatelet and new oral anticoagulants, the use of atazanavir and LPV/r has been discouraged, according to a recent study. In the phase 2/3 randomized, controlled study (NATADEX), 33 patients with mild COVID-19 symptoms are being enrolled to receive either atazanavir or NA-831, a neuroprotective drug, with or without dexamethasone [136].

### 5.3. Translation, Transcription, and Replication Inhibitor

#### 5.3.1. Remdesivir/Veklury

In 2017, Gilead Sciences Company produced remdesivir (GS-5734) as a treatment for SARS-CoV-1, MERS-CoV, hepatitis C, Ebola virus, and Marburg virus infections. It is a monophosphoramidate prodrug that is intracellularly metabolized to an analog of adenosine triphosphate that inhibits viral RNA polymerases. Different studies and trials were performed to evaluate the effectiveness of remdesivir in COVID-19 patients [137–139]; studies declared that remdesivir was superior, shortening the recovery time in adults compared to those who got the placebo [140, 141]. The Solidarity Trial was conducted by the World Health Organization (WHO) and evaluated the efficacy of several drugs for COVID-19 treatment, including remdesivir. The trial included more than 11,000 patients across 30 countries and found that remdesivir had little or no effect on mortality or length of hospital stay [142]. In the other randomized, open-label trial conducted by Gilead Sciences, the efficacy of a 5-day course of remdesivir was evaluated in hospitalized patients with severe COVID-19. The trial found that those who received remdesivir had a significantly higher rate of clinical improvement compared to those who received standard care alone [143]. The Adaptive COVID-19 Treatment Trial-2 (ACTT-2) was conducted by the National Institute of Allergy and Infectious Diseases (NIAID) and evaluated the efficacy of remdesivir in combination with baricitinib (an anti-inflammatory drug) for COVID-19 treatment. The trial included 1,033 hospitalized patients and found that the combination therapy was associated with a shorter time to recovery compared to remdesivir alone [144]. On the other hand, some studies show the ineffectiveness of remdesivir and even report side effects in the recipients of this drug, such as cardiovascular and renal complications [145–147]. Therefore, there is still a need for more comprehensive studies at the national or even global level on patients receiving remdesivir. Finally, on October 22, 2020, the FDA approved or authorized remdesivir for the emergency treatment of adults who weigh at least 40 kg. In laboratory conditions, this drug maintains neutralization activity against the omicron variant and its subvariants [148, 149].

#### 5.3.2. Ribavirin

Ribavirin, a guanosine analog antiviral drug, has been evaluated in COVID-19 patients due to its low cost and high efficacy against SARS-CoV and MERS-CoV [150, 151]. In the multicenter, prospective, open-label study conducted by Hung et al., patients admitted to six hospitals in Hong Kong were randomly assigned to the phase 2 trial. These patients were assigned to a 14-day combination of lopinavir 400 mg and ritonavir 100 mg every 12 h, ribavirin 400 mg every 12 h, and three doses of 8 million international units of interferon beta-1b on alternate days (combination group) or to 14 days of lopinavir 400 mg and ritonavir 100 mg every 12 h (control group). This study indicated the safety of early triple antiviral therapy and its superiority to lopinavir-ritonavir dual therapy in shortening the duration of viral shedding, hospitalization, and improvement of clinical symptoms in patients with mild to moderate COVID-19 [120].

#### 5.3.3. Favipiravir (FPV)

Favipiravir, an antiviral drug and purine nucleic acid analog, inhibits RdRp enzymes and has been effective against bunyavirus, flavivirus, arenavirus, and filovirus [152, 153]. This antiviral drug was used to treat patients infected with influenza and the Ebola virus [154]. Some studies have evaluated favipiravir treatment in patients with COVID-19. Eighty patients with COVID-19 were treated with favipiravir in a nonrandomized trial study, leading to a shorter treatment duration than the control group treated with LPV/r [155]. However, a systematic study that analyzed a total of 2702 studies and 12 clinical trials with 1636 patients found that there was no significant difference in mortality rate and mechanical ventilation requirement between favipiravir treatment and the standard of care in moderate and severe COVID-19 patients [156].

#### 5.3.4. Molnupiravir

Since remedisivir was the primary drug approved by the FDA, due to controversy about its effectiveness, there is a need to focus on finding effective antiviral drugs. One of these drugs is molnupiravir that has recently shown promising results. Molnupiravir is an orally experimental antiviral drug that was initially produced for the treatment of influenza and is effective against many RNA viruses [157, 158]. Molnupiravir inhibits viral RNA polymerases by transforming into a metabolite of ribofuranosyl 5′triphosphate, with a similar mechanism to favipiravir [159, 160]. The results of a phase 1 study of molnupiravir showed that it was safe and tolerable in humans, and phase 2/3 studies confirmed its effectiveness in mild COVID-19 patients, but it was not effective in moderate to severe COVID-19 patients. Interim phase III trial results approved that molnupiravir can reduce the risk of hospital admission or death by 50%, and the UK became the first country to authorize this antiviral for the treatment of mild to moderate patients with at least one risk factor for severe disease [161]. One of the advantages of this drug is that it is taken orally in capsule form. The best time to use it is in the first few days of infection when the body is in a viral phase and has not yet entered the inflammatory phase caused by reactions from the immune system. Mulnupiravir can prevent the disease from progression to severe COVID-19 through virus replication prevention [162]. Molnupiravir increased the frequency of mutations in infected mice, and in the COVID-19 treatment study, the number of these mutations in the combination of the molnupiravir/favipiravir group was significantly higher than molnupiravir alone [163].

#### 5.3.5. Aplidin (Plitidepsin)

Plitidepsin, initially studied for its antitumor properties against multiple myeloma (MM), was rejected twice by the European Medicines Agency as an anticancer drug. However, in 2017, its combination with dexamethasone was authorized in Australia for relapsed/refractory MM patients [164, 165]. In 2017, a combination of plitidepsin and dexamethasone was authorized in Australia for patients with relapsed/refractory MM [166]. Then, in 2020, this combination was examined in COVID-19 patients during the pandemic [167]. The primary intracellular target of plitidepsin could be the eukaryotic elongation factor 1A2 (eEF1A2), which is overexpressed in tumor cells. eEF1A2 is one of the isoforms of eukaryotic translation elongation factor 1 (eEF1A) and is responsible for the enzymatic delivery of aminoacyl tRNAs to the ribosome [168]. This interference ultimately leads to cell cycle arrest, growth inhibition, and the induction of apoptosis through changes in multiple pathways [169]. In vitro studies showed that aplidin affects EF1A, which is an important key to the multiplication and spread of the virus. Therefore, inhibition of it is a strategy for the treatment of viral infection [170]. The efficacy, safety, and toxicity profile of aplidin in COVID-19 patients have been approved in phase I/II clinical study and are moving forward into a phase II/III COVID-19 study [171, 172].

#### 5.3.6. PAXLOVID™ (PF-07321332; Ritonavir)

Ritonavir, packaged with nirmatrelvir (PAXLOVID), is a strong cytochrome P450 (CYP) 3A4 inhibitor and boosts HIV protease inhibitors [173]. Recently, on November 5, 2021, Pfizer reported interim analysis data from a phase II/III clinical trial of an oral antiviral candidate, PAXLOVID™ (PF-07321332; ritonavir), which significantly reduced COVID-19-associated death and hospitalization by 89% compared to placebo in nonhospitalized high-risk patients. PAXLOVID™ prevents virus replication by SARS-CoV-2-3CL main protease (Mpro) inhibition [173]. 3CLpro is an important pivotal role in viral replication, transcription, and its value in the development of antiviral drugs [174]. 1,219 individuals were enrolled in this study, and they got oral doses of either PAXLOVID™ or a placebo every 12 hours for five days. In the overall study population through day 28, only 0.8% of patients who received PAXLOVID™ were hospitalized, and no deaths were reported in these patients compared to 10 (1.6%) deaths and 41 (6.7%) hospitalizations in patients who received placebo [175]. Pfizer plans to submit study data to the U.S. FDA for an emergency use authorization (EUA) instantly. If we want to make a comparison between molnupiravir and PAXLOVID™, we see that the effectiveness of Molnupiravir (50%) is lower than that of PAXLOVID (89%). Molnupiravir is given alone, but PAXLOVID™ is given with ritonavir. The mechanism of molnupiravir is a nucleoside analog, and it induces mutations while the PAXLOVID™ inhibits Mpro and is not mutagenic [176, 177]. But both are still effective against all variants. However, while these two drugs are very promising, more studies are still needed.

### 5.4. Immunomodulators

Inappropriate host immune responses in patients with COVID-19 due to the secretion of proinflammatory cytokines such as IL-1, IL-6, and *t*umor necrosis factor*-α* (TNF*α*) could lead to cytokine storms, organ failure, and severe presentations such as pneumonia and neuropsychiatric symptoms. Therefore, using glucocorticoids, dexamethasone, immunosuppressives, and anti-inflammatory drugs affect the immune cells and could deduct severe presentations and reduce the mortality rate [178, 179].

#### 5.4.1. Steroids

Steroid/corticosteroid such as dexamethasone usage is known as a double-edged sword in fighting against COVID-19 because proper determination of the cumulative dose and duration of corticosteroid therapy is crucial. Survival benefits have been reported for short-term use of it; conversely, long-term use can be harmful [180].

#### 5.4.2. Tocilizumab (Interleukin-6 Receptor Blocker)/Actemra

Tocilizumab is a recombinant monoclonal antibody that inhibits the interleukin-6 (IL-6) receptor, which has been prescribed worldwide despite its unknown efficacy and quality [181]. The efficiency of tocilizumab has been approved for treating rheumatoid arthritis, giant cell arthritis, and Polyarticular juvenile idiopathic arthritis [182, 183]. Tocilizumab therapy in patients with severe COVID-19 resulted in desirable outcomes, and it may be suitable for the treatment of COVID-19 [184–187]. Also, tocilizumab could be efficient in resolving neuropsychiatric symptoms in patients with COVID-19 encephalopathy [188]. On the other hand, some studies have observed different results. It has been shown that tocilizumab therapy could be inefficient in critical patients [189]. Besides, adverse effects such as transient respiratory deterioration, superinfections, and extension of hospital length of stay have been observed [190–192]. The NIH recommends not consuming tocilizumab for patients who do not require ICU-level care, except in clinical trials. Besides, it did not recommend either using or not using tocilizumab for patients within 24 hours of ICU admission who require mechanical ventilation due to insufficient data [193, 194]. In conclusion, tocilizumab may have a positive effect on improving immune status, lung functional injuries, and arterial oxygen saturation, though further precise clinical trial studies are required to determine its efficacy in patients with COVID-19.

#### 5.4.3. Colchicine

Colchicine has been applied thoroughly in numerous cardiovascular medical conditions and some autoinflammatory syndromes [195, 196]. Studies have reported that SARS-CoV-2 can activate the NLR-family pyrin domain-containing 3 (NLRP3) inflammasome. Then, this activation leads to the increase of several inflammatory interleukins (such as IL-1*β* and IL-6) that are responsible for adverse clinical outcomes in COVID-19. One of the important roles of colchicine is to disrupt inflammasome activation [197].

#### 5.4.4. Baricitinib (JAK Inhibitors)

Baricitinib is an immunomodulatory agent which is used in the management and treatment of severe rheumatoid arthritis and is recommended for the treatment of COVID-19-associated pneumonia. This drug prevents COVID-19-induced cytokine storm, thus reducing the extensive inflammation of lung tissue [198]. SARS-CoV-2 invades and enters the cell through endocytosis. AP-2-associated protein kinase 1 (AAK1) and cyclin G-associated kinase (GAK) play significant roles in promoting viral entry in the host cells and the intracellular assembly of viral particles. Baricitinib, as a kinase inhibitor, prevents AAK1 and GAK, thus impeding viral cell entry and internal transport [199, 200]. Another antiviral mechanism of barcitinib is related to its inhibitory effect on IFN signaling [201]. IFN responses are essential for host antiviral defenses. However, recent studies have shown that the expression of ACE2 in human cell lines (including upper airway epithelial cells and primary bronchial cells) is upregulated by type I/II IFN. Baricitinib's suppression of IFN signaling leads to the downregulation of ACE2 and thereby interferes with the ability of SARS-CoV-2 to infect nearby cells [202].

Generally, baricitinib is considered a safe and well-tolerated agent, but it can increase the risk of serious infections and reactive latent infections such as varicella-zoster, herpes simplex, and Epstein-Barr virus strains [31, 203]. Reactivation of latent infections and comorbidities can be significant risk factors in COVID-19 infection control. Therefore, caution is needed when prescribing immunomodulator drugs [204]. Baricitinib has been reported to be associated with bone marrow suppression and hematologic abnormalities, including lymphopenia, anemia, and neutropenia [205, 206]. Indeed, baricitinib should not be used in patients with low neutrophil and lymphocyte counts. Approved doses (2 and 4 mg once daily) have not been indicated to induce anemia in patients with rheumatoid arthritis [207]. Lymphocytopenia has been implicated in the progression of infection in patients with COVID-19 [208]. Therefore, its use requires regular laboratory monitoring. The recommended dose for COVID-19 infection is 2 mg of oral barcitinib evaluated in clinical trials with 10 to 14 days of antiviral therapy [209].

### 5.5. Immunotherapy

Immunotherapeutic has also been suggested as an effective method for the clinical treatment of infectious diseases. Various methods such as plasma therapy, neutralization of monoclonal antibodies, and interferon are studied to strengthen the immune response against SARS-CoV-2.

#### 5.5.1. Convalescent Plasma (CP)

The first successful experience of convalescent blood products dates back to the Spanish influenza pandemic [210]. Passive immunity with convalescent plasma has been established for other types of viruses such as Ebola virus, West Nile Virus, MERS-CoV, SARS-CoV-1, and H1N1, but the efficacy of using convalescent plasma seems to differ depending on the viral agent and prescription protocols [211]. Convalescent plasma can offer an immediate and promising remedy choice at the same time as comparing current drugs and growing new, unique vaccines and therapies. It is necessary to act very quickly. Discovering the donor standards, controlling the blood process and checking out potencies, expanding enough serologic assays for examination, and discovering suitable doses for convalescent plasma are crucial [211]. Overall, observational studies had a more positive view of convalescent therapy than randomized trials. In recent studies, the results of convalescent plasma use have not been satisfactory at all [212, 213], and even in Casadevall et al.'s study conducted in the USA, a strong inverse correlation between CP use and mortality per admission was seen [214].

#### 5.5.2. Neutralizing Monoclonal Antibody

Using neutralizing monoclonal antibodies (nABs) is another approach to the prevention of infectious diseases. nABs are used to bind to one specific target site in the body and cause various changes to enact molecular mechanisms [215]. Viral S protein and glycoprotein are the main target of nABs [216, 217]. As previously mentioned, SARS-CoV-2 and SARS-CoV have a high genetic similarity, especially since both have a similar RBD formation [218]. Therefore, several studies have suggested the use of anti-SARS monoclonal antibodies in patients with COVID-19. Most monoclonal antibodies identified the S1 and RBD fragments of SARS-CoV as 80R, CR3014, CR3022, 68, 201, and 4D4, while some others, such as 1A9, B1, 1F8, and 5E9, recognized the epitopes in the S2 unit of SARS-CoV [219–221]. The combination of CR3014 and CR3022 has been shown as the most effective nABs, which can neutralize RBD and prevent its interaction with ACE2 receptors, thereby reducing viral proliferation and disease severity [222, 223]. On April 16, 2021, the FDA revoked the authorization for emergency usage of the monoclonal antibody bamlanivimab because new SARS-CoV-2 variants showed resistance to it. But alternative monoclonal antibody remains available, including REGEN-COV. On October 10, 2021, the FDA authorized emergency use of REGEN-COV monoclonal antibody (casirivimab and imdevimab, administered together) for COVID-19 patients who are at high risk for progression to severe COVID-19 or for individuals who are not fully vaccinated or whose immune systems cannot respond to the vaccines efficiently [224]. Despite the promising treatment of monoclonal antibodies and their potential as a therapeutic approach and prevention of COVID-19 infection, the large-scale production of monoclonal antibodies, especially against emerging pathogens, is expensive and time-consuming. However, according to the latest update of WHO treatment guidelines, these therapeutic or preventive products are not expected to be effective object on monoclonal antibody resistance variants and subvariants [225, 226].

#### 5.5.3. Interferon- (IFN-) Based Immunotherapy

Expression of interferons (IFNs) as antiviral agents could be an appropriate innate immune response against viral infection. However, inadequate production of IFNs due to viral infection and inhibition of its activity during infection is a fundamental challenge in the immune response to COVID-19 [227]. According to previous evidence regarding IFN-I therapy for MERS-CoV and SARS-CoV infections, type 1 IFNs show high therapeutic capacity in COVID-19 patients [228]. Clinically, the initial combination therapy of SARS-CoV-2 with IFN-*β*1b, lopinavir-ritonavir, was more effective than lopinavir-ritonavir dual therapy. This combination prevented infection and reduced disease progression to severe stages [229, 230]. According to numerous clinical trials, the administration of IFNs in the early stages of COVID-19 prevents the infection from spreading to other cells and triggers a more robust immune response. However, this treatment is not recommended in the acute stage of infection due to the severe immune response and exacerbation of the cytokine storm. According to Zhou et al.'s study, treating patients with IFN-*α*2b, either alone or in combination with Arbidol, reduced the blood levels of the inflammatory markers and also the duration of the detection of the SARS-CoV-2 virus [231]. Based on the results obtained from in vitro and in vivo investigations, IFN could be considered an appropriate pharmaceutical option for COVID-19 patients [232]. Interferon alpha, beta, and lambda have been evaluated for the treatment of COVID-19. Interferon lambda is not currently approved by the FDA for any use [173].

### 5.6. Antibiotics

Since most of the significant COVID-19 mortality related to bacterial co-infection, from the start of the pandemic in addition to antivirals, different kinds of antibiotics had been prescribed, consisting of azithromycin doxycycline, clarithromycin, ceftriaxone, erythromycin, amoxicillin, etc [233]. However, hydroxychloroquine with azithromycin was prescribed as an early remedy for COVID-19, and several studies found no evidence of a benefit of them [234–236]. Also, there have constantly been concerns about antibiotic resistance [237].

### 5.7. Personalized Medicine

One of the emerging and interesting treatments is personalized medicine, the significance of which became more and more apparent with the observation of patients with COVID-19 with various ranges of signs and symptoms from mild to severe.

The purpose of this treatment model is for people with different genetics to take different and particular medications [238]. Evaluating host genetic variants may identify proper targets for therapeutic development [239]. This sort of remedy is still in its infancy, but it seems that the future of medicine is moving in this direction, although large-scale randomized clinical trials with excessive time and cost will be needed [240, 241].

## 6. Vaccine Development

**Figure 3:**
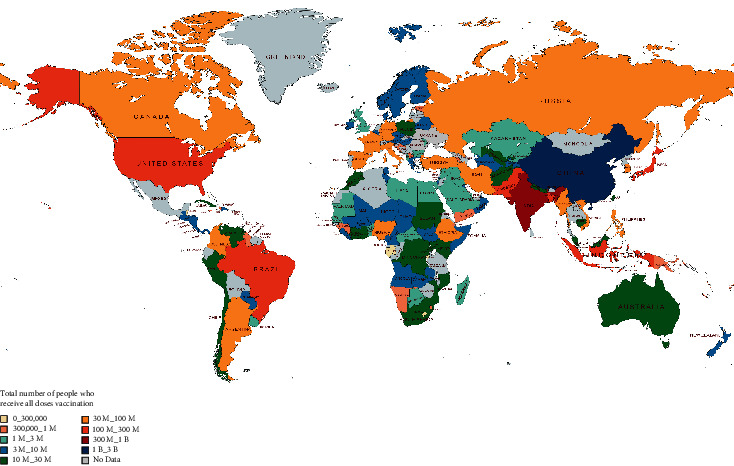
The total number of people who received all doses prescribed by the vaccination protocol (according to the latest 2023 updates of each country).

**Figure 4:**
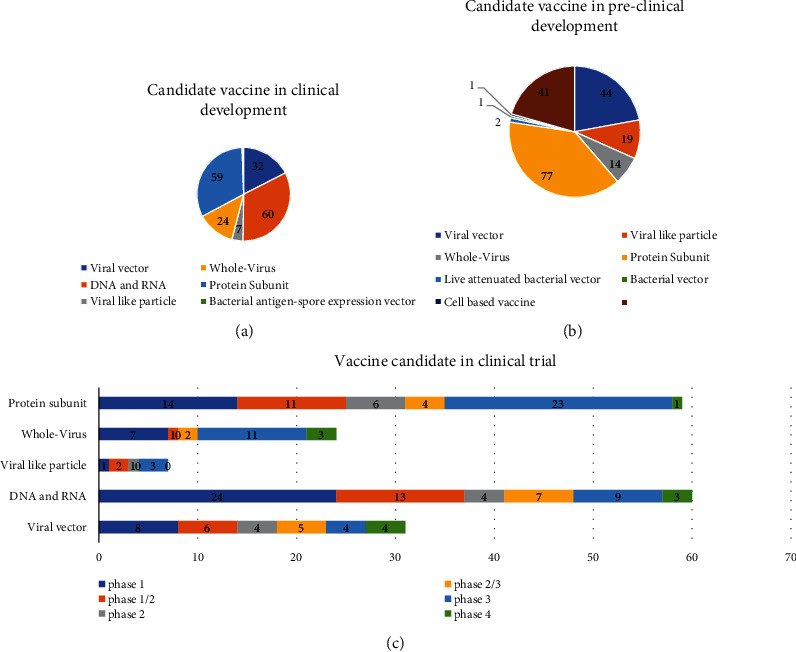
Landscape of COVID-19 vaccine development. Vaccine candidates in the clinical trial (a), preclinical (b), and candidates in the clinical trial phase (c) based on the last update of the WHO report on March 30, 2023.

Globalization, changes in lifestyle and habits, climate change, and traveling lead to emerging infections and increase the occurrence of infections caused by old pathogens such as cholera, Spanish flu, HIV, SARS, Ebola, and Zika [242]. According to WHO weekly report from 12 June 2023, a total of 13,397,334,282 vaccine doses have been administered [243] (Figure 3). The latest WHO report on COVID-19 vaccine products in clinical development on 30 March 2023 stated that 183 vaccines are in clinical development and 199 vaccines are in preclinical development [244] (Figures 4(a)–4(c)). Various protocols were used to prevent the further spread of COVID-19 disease, such as quarantine, isolation of infected individuals, using surface disinfectants and hand sanitizers, supplemental oxygen therapy, and noninvasive ventilation for patients with acute respiratory distress syndrome due to COVID-19. A specific treatment for this disease has not yet been discovered, and developing a specific vaccine for COVID-19 is a high priority for public health [245, 246]. Nowadays, the efficacy and complications of numerous vaccines are being evaluated in randomized clinical trials. The FDA has approved the use of Moderna's coronavirus vaccine, Pfizer/BioNTech, and Janssen's COVID-19 vaccine [247]. These vaccines, developed in the USA, proceed to a phase 3 clinical trial and are now administered intramuscularly. The first vaccine, Pfizer/BioNTech, was approved by the FDA on December 11, 2020, to prevent COVID-19 in individuals aging 16 years and higher. This vaccine is an mRNA vaccine which is used in two dosages with an interval of 21 days [248]. The most common adverse effects following the administration of Pfizer, especially the second dose, include fever, chills, fatigue, headache, muscle pain, and injection site pain [249, 250]. On December 18, 2020, Moderna was approved by the FDA as the second approved vaccine for preventing COVID-19 for use in individuals aging 18 years and higher. Moderna vaccine is an mRNA-1273 vaccine developed by Moderna+National Institute of Allergy and Infectious Diseases (NIAID). This vaccine is administered in two dosages (days 0 and 28) [251, 252]. Common side effects following the administration of the Moderna vaccine, especially after the second dose, usually last several days, including injection site pain, headache, fatigue, chills, muscle pain, nausea and vomiting, and lymph node swelling in the arm to which the vaccine is injected [253]. On February 27, 2021, Janssen was approved by the FDA as the third approved vaccine for COVID-19 prevention in individuals aging 18 years and higher. This vaccine is administered in a single intramuscular dose. Common side effects following administration of this vaccine include injection site reaction, general side effects such as headache, myalgia, nausea, fever, and weakness, and other side effects, including difficulty in breathing, palpitation, dizziness, persistent abdominal pain, and chest pain. Blood clots were seen in some individuals with low platelet counts one to two weeks following the administration of the Janssen vaccine. On 23 April 2021, 15 cases of thrombosis-thrombocytopenia syndrome (TTS) following the administration of Janssen were confirmed; all of them were females aging 18 to 59 years. Following this side effect of the administration of this vaccine, the FDA and CDC recommended stopping the administration of this vaccine [254, 255]. Other vaccines which are used outside the US include AstraZeneca, Sputnik-V, and CanSino which are vector-based vaccines and an inactivated whole-virus SARS-CoV-2 vaccine (by Bharat Biotech and Sinovac) [256, 257]. Here, we summarize the different types of COVID-19 vaccine candidates and clinical trials. Also, some of which are presented in the Supplementary table (available here) [244].

### 6.1. Replicating and Nonreplicating Viral Vectors

Viral vector-based vaccines that contain viruses as vectors to transfer specific antigens to specific receptors or cells are classified into two groups: replicating and nonreplicating vectored vaccines. This classification is based on their ability to replicate in target cells and tissues [258]. Adenoviruses and poxviruses are examples of viral vectors that have both replicating and nonreplicating forms. Replicating viral vectors include measles and vesicular stomatitis. Also, alphaviruses, herpesviruses, and adeno-associated viruses are classified as replication-defective viral vectors [259]. The most viral vectored vaccines are developed to evoke cellular immunity, while broad and potent antibodies can develop through the B cell response following the T helper cell [260]. Adenovirus is the most common viral vector used to develop viral-vectored vaccines [261]. According to the latest report of the WHO on 30 March 2023, a total of 32 COVID-19 vectored vaccines are candidates, which include 25 nonreplicating viral (VVnr) vectors, 4 replicating viral (VVr) vectors, 2 replicating viral vectors + antigen-presenting cell (APC), and one nonreplicating viral vector + antigen-presenting cells. ChAdOx1-S (AZD1222) Covishield is a nonreplicating viral vectored vaccine candidate in clinical development. This vaccine is developed by AstraZeneca and the University of Oxford and is administered in IM form with two dosages (days 0 and 28) [262]. Also, this candidate vaccine proceeds to phase 4 clinical evaluation. On 22 March, the company announced that following the administration of two doses of this vaccine, 79% effective COVID-19 prevention is achieved [263]. Another nonreplicating viral vectored vaccine developed by CanSino Biological Inc./Beijing Institute of Biotechnology is a recombinant novel coronavirus vaccine (adenovirus type 5 vector). This vaccine is in the fourth phase of the clinical trial and is injected as a single IM dose. Another viral-vectored vaccine proceeding to a phase 4 clinical trial is Ad26.COV2.S, also known as Sputnik V, provides a robust protective effect among all participant age groups [264].

#### 6.1.1. nCoV-19 Vaccine (AZD1222)

The period between April 23, 2020, and November 4, 2020, saw 11,636 patients participate in the interim primary efficacy analysis, comprising 7,548 patients in the UK and 4,088 patients in Brazil. Vaccine efficacy was 62.1% in participants who received two standard doses, compared to 90% in participants who received a low dose after a standard dosage. The overall vaccination effectiveness for both groups was 70.4 percent. Ten patients in the control arm were hospitalised for COVID-19 starting 21 days after the first dosage; two of these patients were diagnosed with severe COVID-19, one of which resulted in death. In the 74,341 person-months (median 3–4 months, IQR 1–3–4–8) of safety follow-up, 168 people experienced 175 severe adverse events, compared to 84 in the ChAdOx1 nCoV-19 group and 91 in the control group. Three incidents—one in the ChAdOx1 nCoV-19 group, one in the control group, and one in a participant whose group assignment is yet hidden—were labelled as potentially vaccine-related. An individual who recorded a fever higher than 40°C two days after immunisation in South Africa was reported to have experienced a possible vaccine-related significant adverse event; however, they quickly recovered and did not require hospitalisation. In an interim analysis of ongoing clinical studies, ChAdOx1 nCoV-19 was reported to be effective against symptomatic COVID-19 and to have a tolerable safety profile [265].

#### 6.1.2. Ad5-nCoV Vaccine

Enrollment in the study started in Pakistan, Mexico, Russia, Chile, and Argentina. On January 15, 2021, 150 endpoint cases were met, resulting in the start of the final primary efficacy analysis. At 28 days or more after vaccination, one dose of Ad5-nCoV demonstrated a 57.5% effectiveness against symptomatic, PCR-confirmed COVID-19 infection (21 250 subjects; 45 days median follow-up [IQR 36-58]). There were 36,717 individuals in the primary safety study conducted at the same time as the efficacy analysis, and there was no discernible difference between the case and control groups in terms of the occurrence of serious adverse events. A requested systemic adverse event was reported by 1004 (63%) of 1582 Ad5-nCoV receivers and 729 (46%) of 1572 placebo recipients in the extended safety cohort. Headache was the most frequent of these (699 [44%] of Ad5-nCoV recipients and 481 [30%] of placebo users; p00001). An injection-site adverse event was recorded by 971 (61%) of 1584 Ad5-nCoV recipients and 314 (20%) of 1573 placebo receivers; the most common of these was pain at the injection site, which was reported by 939 (59%) Ad5-nCoV users and 303 (19%) placebo recipients. This study found that healthy persons aged 18 and older can receive one dose of Ad5-nCoV with efficacy and safety [266].

#### 6.1.3. Ad26.COV2.S Vaccine

Ad26.COV2.S is a recombinant, replication-incompetent adenovirus serotype 26 (Ad26) vector encoding a full-length and stabilized SARS-CoV-2 spike protein. In a phase 3 international trial that was randomized, double-blind, and placebo-controlled. 19,630 SARS-CoV-2-negative patients received Ad26.COV2.S, and 19,691 received a placebo, making up the per-protocol population. With an onset at least 14 days after administration (efficacy: 66.9) and at least 28 days after administration (efficacy: 66.1), Ad26.COV2.S protected against moderate-to-severe-critical COVID-19. The effectiveness of the vaccine was greater against severe-critical COVID-19 (onset at 14 days: 76.7%; onset at 28 days: 85.4%). The vaccine's effectiveness against moderate-to-severe-critical COVID-19 with onset at least 14 days and at least 28 days after administration was 52.0% and 64.0%, respectively, and against severe-critical COVID-19 was 73.1% and 81.7%, despite 86 of 91 cases (94.5%) in South Africa with the sequenced virus having the 20H/501Y.V2 variant. Ad26.COV2.S had a higher rate of reactogenicity than the placebo; however, it was typically mild to moderate and momentary. Between the two groups, there was an equal rate of major adverse events. The researchers believed that seven major adverse events in the Ad26.COV2.S group were connected to vaccination. 16 people in the placebo group died, 5 of whom were due to COVID-19, compared to 3 fatalities in the vaccine group (none of which were related to COVID-19) [267].

### 6.2. Messenger RNA(mRNA) Vaccine Candidates

The first successful administration of mRNA-based vaccines was reported in 1993. This mRNA-based vaccine was liposome-encapsulated mRNA encoding the nucleoprotein (NP) of the influenza virus [268]. Following the introduction of intradermal injection of naked mRNA, mRNA-based vaccination was extended in 2000 [269]. mRNA vaccines are produced based on the viral sequence; moreover, lipid nanoparticles are used, allowing direct injection into the host cells' cytoplasm [270]. These vaccines are classified into two groups: self-amplifying mRNA-based vaccines (SAM) and nonamplifying mRNA-based vaccines [271]. Self-amplifying mRNA-based vaccines can rapidly develop with high potency besides being cost-effective, which makes these vaccines suitable for pandemic diseases. As reported by the WHO on 30 March 2023, COVID-19 mRNA-based vaccines are candidates which are presented in the Supplementary Table.

#### 6.2.1. mRNA-1273 SARS-CoV-2: Phase 3 Randomized, Observer-Blinded, Placebo-Controlled Trial

A total of 30,420 volunteers were enlisted in the study, and 15,210 of them were randomized to receive the vaccine or a placebo (15,420 in total). More than 96% of patients received both injections, and 2.2% had a SARS-CoV-2 infection at baseline, either virologically, serologically, or both. 185 participants in the placebo group and 11 people in the mRNA-1273 group were found to have symptoms of COVID-19 sickness; vaccination effectiveness was 94.1%. Key secondary analyses that included assessments 14 days after the first dosage, studies involving participants who showed signs of SARS-CoV-2 infection at baseline, and analyses involving participants 65 years of age or older all revealed comparable efficacy. All 30 people with severe COVID-19—including one fatality—were in the placebo group. The mRNA-1273 group experienced moderate, transitory reactogenicity more frequently after vaccination. Serious adverse events were uncommon and occurred at roughly the same rates in both groups [272].

### 6.3. Virus-Like Particles (VLPs)

Virus-like particles are multiprotein structures that cannot replicate due to their lack of a viral genome [273, 274]. VLP vaccines consist of functional viral proteins responsible for cell-penetrating, hence imitating the organization and combination of the native virus [275]. These vaccines are produced using different expression host systems, including bacteria, mammalian cells, yeast, insect cells, and plant cells. One of the expression host systems for VLPs is bacteria (mainly *E. coli*), accounting for 30% of the VLP vaccine [276, 277]. VLPs are primarily designed to target B cells. Due to its adjuvant properties, both innate and adaptive immune responses will be induced. Using VLP vaccines, the humoral response is activated following the cellular immune response. Prophylactic VLP-based vaccines for hepatitis B commercially available worldwide include Engerix-B and Recombivax, which were approved by the FDA in 1989 and 1986, respectively. Furthermore, prophylactic VLP-based vaccines for human papillomavirus (HPV) which are commercially available worldwide are Gardasil and Cervarix which the FDA approved in 2006 and 2009, respectively [278]. According to the World Health Organization's report on 30 March 2023, there are seven COVID-19 VLP-based vaccines [244].

#### 6.3.1. Coronavirus-Like Particles (CoVLP)

We randomly assigned adults (18 years of age) in a 1 : 1 ratio to receive two intramuscular injections of the CoVLP+AS03 vaccine or a placebo, spaced 21 days apart, in this phase 3, multinational, randomised, placebo-controlled trial. The experiment included 24,141 subjects in total. The effectiveness of the vaccine against any symptomatic COVID-19 induced by the five variations discovered by sequencing was 69.5%. The effectiveness of the vaccine against moderate-to-severe illness was 78.8%, and among participants who were seronegative at baseline, it was 74.0%. There were no severe cases of COVID-19 in the vaccine group, whose median viral load for breakthrough cases was more than 100 times lower than that of the placebo group. Local adverse events occurred in 92.3% and 45.5% of participants, respectively, and systemic adverse events in 87.3% and 65.0% of individuals; these adverse events were mainly mild or moderate, temporary, and happened more frequently in the vaccine group than the placebo group. Up to 21 days after each treatment, the incidence of unexpected adverse events was comparable in the two groups (22.7% and 20.4%), as were the rates from day 43 through day 201 (4.2% and 4.0%) [279].

### 6.4. DNA and RNA Vaccine Candidates

Nucleic acid-based vaccines consist of DNA (plasmid) and RNA (i.e., mRNA). Vaccination through conventional vaccines such as live-attenuated or inactivated vaccines declined the burden of diseases such as measles, tetanus, diphtheria, and polio and also eradicated smallpox. One of the significant problems with conventional vaccination during pandemic situations is its producibility [280]. Two decades ago, DNA-based vaccines were introduced as a new vaccination method that was cost-effective, safe, and needed a short duration for production [281]. Genetic vaccines can be administered in various methods targeting muscle, skin, spleen, nose, and gut surfaces. DNA-based vaccines can be administered in multiple usages such as allergies, autoimmune, infectious diseases, and cancer treatment [282]. The diseases for which DNA-based vaccines are under clinical trial include HIV infection, HBV, and malaria. Due to the specification of DNA vaccines, such as their flexibility, inexpensiveness, ease of production, and no need for cold supply chain storage, they are a good choice for outbreak situations. Genetic vaccines induce adaptive, humoral, and cellular immune response [283]. According to the WHO report on 30 March 2023, 60 COVID-19 nucleic acid-based vaccines are candidates, including 17 DNA-based vaccines and 43 RNA-based vaccines listed in the Supplementary Table. The CVnCoV vaccine is one of the RNA-based vaccine candidates which proceeds to phase 3 in clinical development. This vaccine is developed by CureVac AG and is administered intramuscularly in two dosages (days 0 and 28). The nCov vaccine is one of the DNA-based candidate vaccines which proceeds to phase 3 in clinical development. This vaccine is developed by Cadila Healthcare Ltd. and is administered intradermally in three dosages (days 0, 28, and 56).

#### 6.4.1. SARS-CoV-2 DNA Vaccine INO-4800

INO-4800 was given to 120 healthy individuals without a known history of COVID-19 in a 2-dose regimen (weeks 0 and 4), along with an optional booster dose that could not be given until 8 weeks after dose 2. Six months after the second dose, a persistent antibody response was seen; a homologous booster dose markedly improved immunological responses. In the 2.0 mg dosage group, there was a significant increase in T cells that produce cytokines and in activated CD8+ T cells with lytic capability. There were no treatment-related major adverse events, and INO-4800 seemed to be well tolerated. The majority of adverse reactions were minor, and their incidence did not rise with age or future doses [284].

### 6.5. Whole-Virus Vaccines

All virus vaccines are classified into two groups: inactivated vaccines and live-attenuated vaccines [285]. Inactivated vaccines consist of pathogens without replication and infection but can be immunogen to host cells following their injection, hence stimulating the host immune system. For decades, inactivated vaccines have been used for respiratory disease prevention [286, 287]. Moreover, these vaccines are known as whole killed virus (WKV), which means their viral replication is eliminated by exposure to chemical substances such as formaldehyde, heat, and gamma radiation. WKV vaccines are used to produce enterovirus 71 and influenza virus vaccines. Hanley administered the vaccine to healthy adults in China to evaluate the safety and immunogenicity of an inactivated whole-virus COVID-19. Injection site pain was the most common adverse reaction, while no vaccine-related severe adverse reaction was reported. Also, vaccine-related enhanced respiratory disease (VARED) has not been seen [288].

Live-attenuated vaccines originated from combating smallpox in China. These vaccines contain a weakened version of the virus. They stimulate a robust immune response and lead to a long-lasting immunological memory and reactogenicity resembling natural infection in host cells. At the same time, inactive vaccines are less reactogenic and lead to less immune response, then multiple doses of WKV vaccines should be injected [288, 289]. The most beneficial live-attenuated vaccine is Bacillus Calmette-Guerin (BCG) [289]. According to the WHO report on 30 March 2023, 24 COVID-19 whole virus vaccines are candidates, including 22 inactivated virus vaccines and 2 live-attenuated virus vaccines. Seven inactivated virus candidate vaccines proceed to phase 3 clinical evaluations administered intramuscularly in two dosages. Inactivated SARS-CoV-2 vaccine (Vero cell) and CoronaVac which are developed by Sinopharm + China National Biotec Group Co + Beijing Institute of Biological Products and Sinovac Research and Development Co., Ltd., respectively, proceed to phase 4 clinical trial and are administered intramuscularly in two dosages.

#### 6.5.1. QazCovid-in

A multicenter, randomised, single-blind, placebo-controlled phase 3 efficacy trial with a 180-day follow-up period was conducted in three clinical centres in Kazakhstan from December 25, 2020, to July 11, 2021, to assess the efficacy of the whole virion formaldehyde-inactivated anti-COVID-19 vaccine QazCovid-in. After two intramuscular immunisations, this vaccine was safe for the whole six-month observation period, causing only localised, transient side effects. Participants' concurrent illnesses had no impact on the vaccine's safety. Out of 2400 participants who received the vaccine, 31 were diagnosed with COVID-19; 600 placebo subjects had 43 incidences of COVID-19, which began 14 days after the first dose during the 180-day observation period. Only one severe COVID-19 case was found in a vaccine recipient who also had chronic heart failure as a coexisting condition. Within the 180-day observation period, the QazCovid-in vaccine's protective effectiveness reached 82.0% [290].

#### 6.5.2. CoronaVac Vaccine

CoronaVac, also called Sinovac COVID-19, is a two-dose SARS-CoV-2 vaccine that has been adjuvanted with aluminium hydroxide and inactivated with propiolactone (BPL) [291]. A phase 3 clinical trial including 12,688 participants was carried out in Brazil between July 21 and December 16, 2020, to assess the effectiveness and safety of CoronaVac-vaccinated healthcare personnel who treated patients with COVID-19. The vaccination or placebo was administered to each subject at least once. 9823 patients received both doses out of this total. The outcomes showed that the vaccine's effectiveness against hospitalisation and symptomatic COVID-19 was 50.7% and 100%, respectively. Most adverse reactions were mild to severe, and the most frequent ones were myalgia, headaches, discomfort at the injection site, and exhaustion. There were also only a few allergy reactions, all of which were grade 1 or 2 [292]. In Turkey, 10,218 people between the ages of 18 and 59 who had no prior history of COVID-19 and had negative real-time polymerase chain reaction (PCR) findings participated in another phase 3 clinical trial of CoronaVac. Participants either received the vaccination or a placebo on days 0 and 14. The immunisation group observed nine real-time PCR-confirmed COVID-19 cases two weeks after receiving the second dose of the vaccine, whereas the placebo group observed 32 cases over the course of the 43-day follow-up period. The efficacy of the vaccine was reported to be 83%. The frequency of adverse reactions was 18.9% in the vaccine group and 16.9% in the placebo group, with no fatalities or grade 4 side events. Fatigue was the most frequent systemic adverse effect, occurring in 8.2% of vaccination recipients and 7% of placebo recipients, respectively [293].

### 6.6. Protein-Based Vaccines

Coronavirus belongs to the Coronaviridae family and the Nidovirales order; it is described as a single-stranded RNA virus with four structural proteins, including E, M, N, and S proteins which play an essential role in obliterating the body's immune response in infected individuals [2, 294]. The S protein is composed of two functional subunits: S1 and S2. S1 has been cited for binding to ACE2 in the human body [295]. ACE2 is a protein found in the lungs, liver, heart, and kidneys. Furthermore, the S2 subunit is responsible for membrane fusion [296, 297]. These features led to the development of protein-based vaccines that trigger antibodies to prevent the virus from binding to ACE2 and membrane fusion, hence neutralizing the infection of the COVID-19 virus [295, 298]. Due to the need to penetrate the membrane and bind to human receptors for the virus's life cycle, the S protein is an essential target for developing COVID-19 vaccines. According to the WHO report on 30 March 2023, a total of 59 COVID-19 protein subunit-based vaccines are candidates. SARS-CoV-2 rS/Matrix M1-Adjuvant (full-length recombinant SARS-CoV-2 glycoprotein nanoparticle vaccine adjuvanted with matrix M) is one of the protein subunit-based vaccines is a candidate in clinical development. This vaccine is developed by Novavax and is administered intramuscularly in two dosages (days 0 and 21). Also, this candidate vaccine proceeds to a phase 3 clinical trial. Another protein subunit-based vaccine that is a candidate in clinical development and proceeding to a phase 3 clinical trial is the recombinant SARS-CoV-2 vaccine (CHO Cell). This vaccine is developed by the Anhui Zhifei Longcom Biopharmaceutical + Institute of Microbiology, Chinese Academy of Sciences, and is administered intramuscularly in 2 (days 0 and 28) or 3 (days 0, 28, and 56) dosages. Other protein subunit vaccine candidates are listed in the Supplementary Table.

#### 6.6.1. Nanocovax

A full-length prefusion stabilised recombinant SARS-CoV-2 spike glycoprotein vaccine for the 2nd subunit of the coronavirus that causes severe acute respiratory syndrome (SARS-CoV-2), along with aluminium hydroxide adjuvant. 13,007 individuals who were 18 years of age or older participated in this clinical trial phase 3. The frequency of both requested and unexpected adverse events (AE) was comparable across the vaccination and placebo groups up until day 180. Both the vaccination and placebo groups experienced 100 severe adverse events (SAE). 100 SAEs in all, 96 of which were found to be unrelated to the experimental products. According to the Data and Safety Monitoring Board (DSMB) and investigators, 4 SAEs may have been connected. In the majority of subjects, reactogenicity was nonexistent or minimal and only lasted a brief time. These results emphasise Nanocovax's outstanding safety record. In terms of immunogenicity, Nanocovax elicited significant IgG and neutralising antibody responses. Importantly, on day 42, neutralising antibody titers and anti-S-IgG levels were higher than they were in instances of naturally occurring infections. Instead of Th1, Nanocovax was found to cause Th2 polarisation. The vaccine's effectiveness against illness with symptoms was 51.5%. 93.3% of people had VE against death and serious illness. Notably, the delta variant strain predominated during the time of our study [299].

#### 6.6.2. SpikoGen

A subunit coronavirus disease 2019 (COVID-19) vaccine made of an Advax-CpG55.2TM adjuvant and recombinant coronavirus 2 spike protein for severe acute respiratory illness. In all, 12,657 and 4219 participants were randomly assigned to the SpikoGen® and placebo groups, and after receiving the second dosage for 14 days, they were monitored for a median of 55 days (interquartile range, 48-60 days) and 51 days (interquartile range, 46-58 days), respectively. In the final per-protocol analysis, the SpikoGen® group had 247 of 9998 (2.4%) COVID-19 instances, while the placebo group had 119 of 3069 (3.8%) cases. This translated to a 43.99% vaccination effectiveness. The computed effectiveness for all participants who got both doses was 44.22%. The vaccine's effectiveness against severe disease was 77.51% as of 2 weeks following the second dosage, when 5 of 9998 (0.05%) participants in the SpikoGen® group and 6 of 3069 (0.19%) individuals in the placebo group both developed severe COVID-19. The SpikoGen® vaccination received favourable reviews [300].

## 7. Vaccines Effectiveness against VOCs

**Figure 5:**
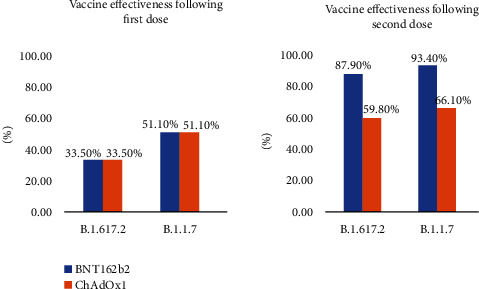
Comparing the effectiveness of BNT162b2 and ChAdOx1 nCoV-19 vaccines against delta variants after the first and second dose of vaccination.

Following the emergence and global spread of SARS-CoV-2 variants, public health concern is about the effect of new variants on the efficacy of the vaccines. Here are some studies which evaluate the vaccine's effectiveness on new SARS-CoV-2 variants. In the study carried out by Bernal et al., they assess the effectiveness of BNT162b2 and ChAdOx1 nCoV-19 vaccines against delta variants in England. They noticed lower vaccine effectiveness in B.1.617.2 cases compared to B.1.1.7 cases following the first and second doses of vaccination [301] (Figure 5).

The study, which was done among vaccinated healthcare professionals in Finland to evaluate vaccine-induced immunity, indicated a very high level of antibody induction against viral spike protein and high titers of neutralizing antibodies following two-dose vaccination with the BNT162b2 mRNA COVID-19 vaccine. Also, this study illustrated approximately good vaccine effectiveness against new variants with good cross-reactivity to D614G and B.1.1.7 variants in all vaccines and detectable neutralizing antibodies to the B.1.351 variant in 92% of the vaccines [302].

**Figure 6:**
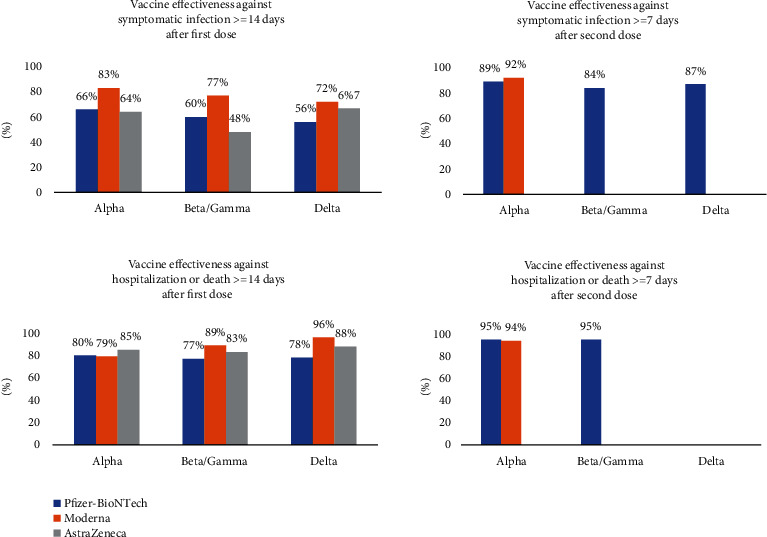
Comparing the effectiveness of Pfizer-BioNTech, Moderna, and AstraZeneca vaccines against variants of concern (VOCs) after the first and second dose of vaccination.

The study, which was done in Ontario, Canada, assessed the effectiveness of Pfizer-BioNTech, Moderna, and AstraZeneca vaccines against new variants of concern (VOCs). The result showed the good to excellent effectiveness of these three vaccines following a single dose and higher efficacy following a second dose against symptomatic infection and severe outcomes caused by new variants of concerns [303] (Figure 6).

**Figure 7:**
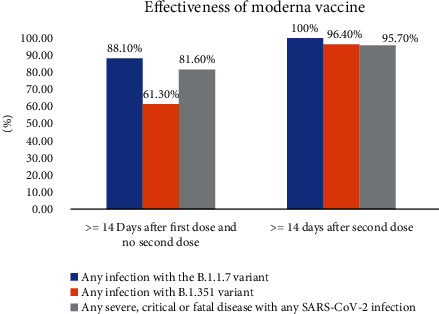
Effectiveness of Moderna vaccine against the B.1.1.7 and B.1.351 variants after the first and second dose of vaccination.

In the study conducted by Barros-Martins et al. among Hannover Medical School healthcare professionals, they indicated the induction of significantly higher frequencies of spike-specific CD4+ and CD8+ T cells and high titers of neutralizing antibodies against the B.1.1.7, B.1.351, and P.1 VOCs by BNT162b2 in comparison with the ChAdOx1-nCov-19 vaccine [304]. The study which was done in Qatar evaluated the mRNA-1273 (Moderna) vaccine's effectiveness against the B.1.1.7 and B.1.351 variants. They concluded the high effectiveness of Moderna against B.1.1.7 and B.1.351 symptomatic and asymptomatic infections and severe outcomes, even after a single dose [304] (Figure 7).

## 8. Conclusion

We are eagerly awaiting the discovery of a safe method of overcoming COVID-19. Also, we speculate that the current emergency requires massive studies on the coronaviruses, their mechanism of infection, and reapproaching to developing drugs to overcome the current and prevent a future pandemic. Due to the financial burden and rates of COVID-19, most public health concerns are related to this pandemic, and different nations are dealing with restricting the transmission of this virus. In order to deal with this challenge, they adopted many solutions such as quarantine, social distancing, wearing masks, hand sanitization, and surface cleaning and disinfecting. One of the essential strategies to prevent the spread of the virus is using vaccines. Up to now, 128 vaccines have reached the clinical trial stage, and 194 vaccines are in the preclinical phase. Due to the passage of time for public vaccination in many countries and the emergence of new variants of this virus, one of the public concerns is the use of booster doses. Due to the few studies that have been done in the field of booster dose effectiveness, more studies and research are needed in this field. Note that besides the efficacy and safety of the vaccines, global access to the vaccine is critically important for vaccine coverage all over the world to fight the pandemic. Nowadays, healthcare providers utilize some medications which can be potentially effective against COVID-19. The medications above should be administered until discovering a SARS-CoV-2-specific drug. Furthermore, many pharmaceutical companies make great efforts to discover and produce effective specific drugs to combat COVID-19 in parallel with vaccination.

## Figures and Tables

**Table 1:** SARS-CoV-2 variants of interest (VOI) [49].

SARS-CoV-2 variants nomenclature				First identification	Current status	Date of designation
WHO label	Pango lineages	GISAID clade	Nextstrain clade			
Epsilon	B.1.427 and B.1.429	GH/452R.V1	21C	United States of America	VBM	VOI: 5-Mar-2021 Previous VOI: 6-Jul-2021

Zeta	P .2	GR/484K.V2	20B/S.484K	Brazil	VBM	VOI: 17-Mar2021 Previous VOI: 6-Jul-2021

Eta	B.1.525	G/484K.V3	21D	Multiple countries	VBM	VOI: 17-Mar2021 Previous VOI: 20-Sep-2021

Theta	P.3	GR/1092K.V1	21E	Philippines		VOI: 24-Mar2021 Previous VOI: 6-Jul-2021

Iota	B.1.526	GH/253G.V1	21F	United States of America	VBM	VOI: 24-Mar-2021 Previous VOI: 20-Sep-2021

Kappa	B.1.617.1	G/452R.V3	21B	India	VBM	VOI: 4-Aprl-2021 Previous VOI: 20-Sep-2021

Lambda	C.37	GR/452Q.V1	21G	Peru		VOI: 14-Jun-2021 Previous VOI: 9-Mar-2022

Mu	B.1.621	GH	21H	Colombia	VBM	VOI: 30-Aug-2021 Previous VOI: 9-Mar-2022

VOI: variants of interest; VOC: variants of concern; VBM: variant being monitored.

**Table 2:** SARS-CoV-2 variants of concern (VOC) [49].

SARS-CoV-2 variants nomenclature				First identification	Current status	Date of designation
WHO label	Pango lineages	GISAID clade	Nextstrain clade			
Alpha	B.1.1.7	GRY (formerly GR/501Y.V1)	20I (V1)	United Kingdom	VBM	VOC: 18-Dec-2020

Beta	B.1.351 B.1.351.2 B.1.351.3	GH/501Y.V2	20H (V2)	South Africa	VBM	VOC: 18-Dec-2020

Gamma	P.1 P.1.1 P.1.2	GR/501Y.V3	20J (V3)	Brazil	VBM	VOC: 11-Jan-2021 Previous VOC: 09-Mar-2022

Delta	B.1.617.2 AY.1 AY.2 AY.3	G/478K.V1	21A	India	VBM	VOI: 4-Apr-2021 VOC: 11-May-2021

Omicron parent lineage	B.1.1.529	GR/484A	21k	Multiple countries	VOC	VOC: 26-Nov-2021 Previous VOC: 14-Mar-2023

VOI: variants of interest; VOC: variants of concern; VBM: variant being monitored.

**Table 3:** Drugs with antiviral efficacy on SARS-CoV-2.Please confirm the rearranged sequence of table citations in text as they should be in ascending sequence as per journal style.

Classification	Agent	The main usage	Mechanism of action against COVID-19	Ref.
Viral enter and fusion	Chloroquine/hydroxychloroquine	Malaria and autoimmune disease	Interferes with glycosylation of ACE2, proteolytic processing, and endosomal acidification	[231]
	Arbidol-umifenovir	Influenza, SARS	Inhibition of S-protein-ACE2 prevents viral entry into the host cell fusion inhibition	[99]
	Ivermectin	HIV, dengue, influenza, Zika virus, yellow fever virus, etc.	Imped the attachment of SARS-CoV-2 to the host cell, inhibit importin alpha/beta-1 nuclear transport proteins	[102, 103]

Viral protease inhibitor	Lopinavir/ritonavir	HIV, SARS, MERS	Inhibit the viral 3CL protease—inhibit viral replication and release from the cell	[305]
	Darunavir	HIV	Inhibiting one of the vital main protease enzyme	[122]
	Nelfinavir	HIV	Binds to the protease active site and inhibits the activity of the enzyme Inhibit viral entry via the angiotensin-converting enzyme 2 (ACE2) receptor and transmembrane serine protease 2	[127]
	Atazanavir	HIV	Inhibit the viral 3CL protease	[132]

Transcription and replication inhibitor	Remdesivir	HBV, HCV, Ebola, Marburg virus, HIV	Interferes with viral RNA-dependent RNA polymerase (RdRp) and reduces viral RNA synthesis to arrest viral replication	[231]
	Ribavirin	RSV	Inhibits viral RNA polymerase	[151]
	Favipiravir (FPV)	Viral diseases	Inhibits viral polymerase and replicase activity	[155]
	Aplidin	Antitumor, antiviral	Inhibits the human protein eEF1A which has potential interactions with multiple coronavirus proteins and inhibits viral translation	[167]
	Molnupiravir	Influenza	Impede replication by inducing RNA mutations	[176]
	PAXLOVID	SARS-CoV-2	Inhibit the viral 3CL protease—inhibit viral replication—not mutagenic	

Immunomodulators	Steroids	HLH, ARDS	Immunosuppressive and anti-inflammatory	[306]
	Tocilizumab	Rheumatic diseases	Blocking IL-6 signalling and its mediated inflammatory response	[187]
	Colchicine	Treat gout and rheumatic disease	Combat the hyperinflammatory state of COVID-19 (e.g., cytokine storm) by suppressing proinflammatory cytokines and chemokines	[307]
	Baricitinib	SARS	Inhibiting the cytokine storm and JAK 1 and 2 enzymes	[198]

## Data Availability

The authors confirm that the data supporting the findings of this study is available within the article.

## Abbreviations

FDA: Food and Drug Administration
E: Envelope
M: Membrane
N: Nucleocapsid
S: Spike
SARS-CoV-2: Severe acute respiratory syndrome coronavirus 2
WHO: World Health Organization
NAAT: Nucleic acid amplification test
LFI: Lateral flow immunoassays
ORF: Open reading frame
Nsp: Nonstructural proteins
RdRp: RNA-dependent RNA polymerase
ACE2: Angiotensin-converting enzyme 2
CTD: C-terminal domain
NTD: N-terminal domain
RBD: Receptor-binding domain
FP: Fusion peptide
IFP: Internal fusion peptide
TM: Transmembrane domain
TMPRSS: Transmembrane protease serine protease
PP: Polypeptides
NF-*κ*B: Nuclear factor kappa B
TNF*α*: *T*umor necrosis factor*-α*
IL: Interleukin
IFNs: Interferons
ER: Endoplasmic reticulum
ERGIC: ER-Golgi compartment
CQ/HCQ: Chloroquine/hydroxychloroquine
ARB: Arbidol
LPV/r: Lopinavir/ritonavir
NIH: National Institutes of Health
AAK1: AP-2 associated protein kinase 1
GAK: G-associated kinase
nABs: Neutralizing monoclonal antibodies
NIAID: National Institute of Allergy and Infectious Diseases
TTS: Thrombosis-thrombocytopenia syndrome
VVnr: Nonreplicating viral vectored
VVr: Replicating viral vectored
APC: Antigen-presenting cell
mRNA: Messenger RNA
NP: Nucleoprotein
VLPs: Virus-like particles
WKV: Whole killed virus
VARED: Vaccine-related enhanced respiratory disease
BCG: Bacillus Calmette-Guerin
GISAID: Global initiative on sharing all influenza data
VOI: Variants of interest
VOC: Variants of concern.

## Supplementary Materials

Supplementary Materials The supplementary material included vaccine development, vaccine candidate, vaccination methods, and clinical stage.
