# Supplementary material for: COVID-19: An Overview of SARS-CoV-2 Variants—The Current Vaccines and Drug Development
Source: Biomed Res Int. 2023 Aug 29;2023:1879554. doi: 10.1155/2023/1879554 (PMC10480030; doi:10.1155/2023/1879554)
Supplement: Supplementary Materials — The supplementary material included vaccine development, vaccine candidate, vaccination methods, and clinical stage. [file 1879554.f1.pdf]

Supplementary Table: Vaccine development [1].

| Vaccine candidate | Vaccine platform | Developer | Dosage | Vaccination methods | Clinical stage |
|---|---|---|---|---|---|
| **Viral Vectored Vaccine** | | | | | |
| **ChAdOx1-S - (AZD1222) Covishield Vaxzevria** | Viral vector (Non-replicating) | AstraZeneca + University of Oxford | 1-2 (Day0+28) | IM | 4 |
| **Recombinant novel coronavirus vaccine (Adenovirus type 5 vector) Ad5-nCoV** | Viral vector (Non-replicating) | CanSino Biological Inc./Beijing Institute of Biotechnology | 1 (Day0) | IM | 4 |
| **Recombinant COVID-19 vaccine (adenovirus type 5 vector) for Inhalation (Ad5-nCoV-IH)** | Viral vector (Non-replicating) | CanSino Biological Inc./Beijing Institute of Biotechnology | 1 (Day0) | IH | 4 |
| **Gam-COVID-Vac Adeno-based (rAd26-S+rAd5-S) Sputnik V COVID-19 vaccine** | Viral vector (Non-replicating) | Gamaleya Research Institute ; Health Ministry of the Russian Federation | 2 (Day0+21) | IM | 3 |
| **Ad26.COV2.S** | Viral vector (Non-replicating) | Janssen Pharmaceutical | 1-2 (Day0 or Day0+56) | IM | 4 |
| **GRAd-COV2 (Replication defective Simian Adenovirus (GRAd) encoding S)** | Viral vector (Non-replicating) | ReiThera + Leukocare + Univercells | 1 (Day0) | IM | 2/3 |
| **VXA-CoV2-1 Ad5 adjuvanted Oral Vaccine platform** | Viral vector (Non-replicating) | Vaxart | 2 (Day0+28) | Oral | 2 |
| **MVA-SARS-2-S** | Viral vector (Non-replicating) | University of Munich (Ludwig-Maximilians) | 2 (Day0+28) | IM | 1 |
| **V591-001 - Measles-vector based (TMV-o38)** | Viral vector (Replicating) | Merck & Co. + Themis + Sharp & Dohme + Institute Pasteur + Univeristy of Pittsburgh | 1-2 (Day0+28) | IM | 1/2 |

| | | | | | |
|---|---|---|---|---|---|
| **DelNS1-2019-nCoV-RBD-OPT1 (Intranasal flu-based-RBD )** | Viral vector (Replicating) | University of Hong Kong, Xiamen University and Beijing Wantai Biological Pharmacy | 2 (Day0+28) | IN | 3 |
| **Covid-19/aAPC vaccine. The Covid-19/aAPC vaccine is prepared by applying lentivirus modification with immune modulatory genes and the viral minigenes to the artificial antigen presenting cells (aAPCs).** | Viral vector (Replicating) + APC | Shenzhen Geno-Immune Medical Institute* | 3 (Day0+14+28) | SC | 1 |
| **LV-SMENP-DC vaccine. Dendritic cells are modified with lentivirus vectors expressing Covid-19 minigene SMENP and immune modulatory genes. CTLs are activated by LV-DC presenting Covid-19 specific antigens.** | Viral vector (Non-replicating) + APC | Shenzhen Geno-Immune Medical Institute* | 1 (Day0) | SC & IV | 1/2 |
| **Human Adenovirus Type 5: hAd5 S+N vaccine (S-Fusion + N-ETSD). E2b-Deleted Adeno.** | Viral vector (Non-replicating) | ImmunityBio, Inc | 1-2 (Day0+21) | SC | 1/2 |
| **COH04S1 (MVA-SARS-2-S) - Modified vaccinia ankara (sMVA) platform + synthetic SARS-CoV-2** | Viral vector (Non-replicating) | City of Hope Medical Center + National Cancer Institute | 1-2 (Day0+28) | IM | 2/3 |
| **rVSV-SARS-CoV-2-S Vaccine** | Viral vector (Replicating) | Israel Institute for Biological Research | 1 (Day0) | IM | 2/3 |
| **Dendritic cell vaccine AV-COVID-19. A vaccine consisting of autologous dendritic cells loaded** | Viral vector (Replicating) + APC | Aivita Biomedical, Inc. | 1 (Day0) | IM | 2 |
| **AdCLD-CoV19 (adenovirus vector)** | Viral vector (Replicating) | Cellid Co.,Ltd.* | 1 (Day0) | IM | 2 |
| **AdCOVID, Adenovirus-based platform expresses receptor-binding domain (RBD) of spike protein** | Viral vector (Non-replicating) | Altimmune, Inc. | 1-2 (Day0) | IN | 1 |

| | | | | | |
|---|---|---|---|---|---|
| **BBV154, Adenoviral vector COVID-19 vaccine** | Viral vector (Non-replicating) | Bharat Biotech International Limited | 1 (Day0) | IN | 3 |
| **Modified Vaccinia Virus Ankara (MVA) vector expressing a stabilized SARS-CoV-2 spike protein** | Viral vector (Non-replicating) | German Center for Infection Research | 2 (Day0+28) | IM | 1/2 |
| **Chimpanzee Adenovirus serotype 68 (ChAd) and self-amplifying mRNA (SAM) vectors expressing spike alone, or spike plus additional SARS-CoV-2 T cell epitopes.** | Viral vector (Non-replicating) | Gritstone Oncology | 2-3 (Day 0+14+28 or 0+28+56 or 0+112) | IM | 1 |
| **COVIVAC. Newcastle Disease Virus (NDV) expressing membrane-anchored pre-fusion-stabilized** | Viral vector (replicating) | Institute of Vaccines and Medical Biologicals, Vietnam | 2 (Day 0+28) | IM | 1/2 |
| **SC-Ad6-1, Adneviral vector vaccine** | Viral vector (Non-replicating) | Tetherex Pharmaceuticals Corporation | 1-2 (Day 0 +/- 21) | IM | 2 |
| **PIV5 vector that encodes the SARS-CoV-2 spike protein** | Viral vector (Non-replicating) | CyanVac LLC | 1 (Day 0) | IN | 2 |
| **AZD2816; adenoviral vector ChAdOx platform and based on the Beta (B.1.351) variant** | Viral vector (Non-replicating) | AstraZeneca + University of Oxford | 2 (Day0+28) | IM | 2/3 |
| **AAV5-RBD-S vaccine (BCD-250), A recombinant Adenovirus-Associated viral Vector (AAV-5) encoding spike protein** | Viral vector (Non-replicating) | Biocad | 1 (Day0) | IM | 1/2 |
| **Ad5-triCoV/Mac or ChAd-triCoV/Mac, new experimental adenovirus-based vaccines expressing SARS-CoV-2 spike, nucleocapsid and RNA polymerase proteins** | Viral vector (Non-replicating) | McMaster University | 1 (Day0) | AE | 1 |
| **Ad26.cov2.s+bcg vaccine. AD26-BCG** | Viral vector (Non-replicating) | Han Xu, M.D., Ph.D., FAPCR, Sponsor-Investigator, IRB Chai | 1 (Day0) | ID | 1 |

| | | | | | |
|---|---|---|---|---|---|
| **NDV-HXP-S; A Live Recombinant Newcastle Disease Virus-vectored COVID-19 Vaccine** | Viral vector (Replicating) | Sean Liu, Icahn School of Medicine at Mount Sinai | 1 (Day0) | IN | 2/3 |
| **MVA-SARS-2-ST Vaccine** | Viral vector (Non-replicating) | Hannover Medical School | 1 (Day0) | IH | 1 |
| **Convidecia Vaccine (Ad5-nCoV). Bivalent Recombinant COVID-19 Vaccine (Adenovirus Type 5 Vector)** | Viral vector (Non-replicating) | CanSino Biologics Inc. | 2 (Day 0+21) | IM | 3 |
| **CoVacHGMix adenoviral vector vaccine** | Viral vector (Non-replicating) | Ankara City Hospital Bilkent | 2 (Day 0+28) | IM | 1 |
| **H3N2 recombinant attenuated influenza vector with modified NS gene coding for the N protein fragment of SARS-CoV-2** | Viral vector (Non-replicating) | Research Institute of Influenza | 2 (Day 0+21) | IN | 1/2 |
| **Recombinant COVID-19 Vaccine (Adenovirus Vector)** | Viral vector (Non-replicating) | Wuhan BravoVax | 1 (Day0) | IN | 1 |
| **COVID19 Oral Vaccine Consisting of Bacillus Subtilis Spores** | BacAg-SpV -Bacterial antigen-spore expression vector | DreamTec Research Limited | 3 (Day0+14+28) | Oral | NA |
| **DNA and RNA vaccine** | | | | | |
| **CVnCoV Vaccine** | RNA based vaccine | Curevac AG | 2 (Day0+28) | IM | 3 |
| **mRNA-1273 (Spikevax )** | RNA based vaccine | Moderna + National Institute of Allergy and Infectious Diseases (NIAID) | 2 (Day0+28) | IM | 4 |
| **BNT162b2 (3 LNP-mRNAs ), also known as "Comirnaty"** | RNA based vaccine | Pfizer/BioNTech + Fosun Pharma | 2 (Day0+21) | IM | 4 |
| **INO-4800+electroporation** | DNA based vaccine | Inovio Pharmaceuticals + International Vaccine Institute + Advaccine | 2 (Day0+28) | ID | 3 |
| **AG0301-COVID19** | DNA based vaccine | AnGes + Takara Bio + Osaka University | 2 (Day0+14) | IM | 2/3 |
| **nCov vaccine** | DNA based vaccine | Zydus Cadila | 3 (Day0+28+56) | ID | 3 |
| **GX-19** | DNA based vaccine | Genexine Consortium | 2 (Day0+28) | IM | 2/3 |

| | | | | | |
|---|---|---|---|---|---|
| **ARCT-021** | RNA based vaccine | Arcturus Therapeutics | NR | IM | 2 |
| **LNP-nCoVsaRNA** | RNA based vaccine | Imperial College London | NR | IM | 1 |
| **SARS-CoV-2 mRNA vaccine (ARCoV)** | RNA based vaccine | Academy of Military Science (AMS), Walvax Biotechnology and Suzhou Abogen Biosciences | 2 (Day0+14) Or (Day0+28) | IM | 3 |
| **Covigenix VAX-001 - DNA vaccines + PLV formulation** | DNA based vaccine | Entos Pharmaceuticals Inc. | 2 (Day0+14) | IM | 1 |
| **CORVax - S Protein Plasmid DNA Vaccine** | DNA based vaccine | OncoSec Immunotherapies; Providence Health & Services | 2 (Day0+14) | ID | 1 |
| **bacTRL-Spike oral DNA vaccine** | DNA based vaccine | Symvivo Corporation | 1 (Day0) | Oral | 1 |
| **GLS-5310** | DNA based vaccine | GeneOne Life Science, Inc.* | 2 (Day0+56) Or (Day0+84) | ID | 1/2 |
| **COVIGEN** | DNA based vaccine | University of Sydney, Bionet Co., Ltd | 2 (Day0+28) | ID | 1 |
| **COVID-eVax, a candidate plasmid DNA vaccine of the Spike protein** | DNA based vaccine | Takis + Rottapharm Biotech | 2 ( Day0+28) | IM | 1/2 |
| **EXG-5003; a temperature-sensitive self-replicating RNA vaccine expressing the receptor binding domain of the SARS-CoV-2 spike protein.** | RNA based vaccine | Elixirgen Therapeutics, Inc | 1 (Day0) | ID | 1/2 |
| **LNP-nCOV saRNA-02 vaccine; Self-amplifying RNA (saRNA) encapsulated in lipid nanoparticles (LNP)** | RNA based vaccine | MRC/UVRI and LSHTM Uganda Research Unit | 2 ( Day0+28) | IM | 1 |
| **AG0302-COVID19** | DNA based vaccine | AnGes, Inc | 2-3 (Day0+14+28) | IM | 1/2 |

| | | | | | |
|---|---|---|---|---|---|
| **Plasmid DNA vaccine SCOV1 + SCOV2. COVIDITY** | DNA based vaccine | Scancell Ltd | 2<br>( Day0+28) | ID | 1 |
| **VB10.2129, a DNA plasmid vaccine, encoding the receptor binding domain (RBD)** | DNA based vaccine | Vaccibody AS | 1-2<br>( Day0+21) | IM | 1/2 |
| **VB10.2210, DNA plasmid vaccine, encodes multiple immunogenic and conserved T cell epitopes spanning multiple antigens across the SARS-CoV-2 genome** | DNA based vaccine | Vaccibody AS | 1-2<br>( Day0+21) | IM | 1/2 |
| **SARS-CoV-2 DNA vaccine (delivered IM followed by electroporation)** | DNA based vaccine | The University of Hong Kong; Immuno Cure 3 Limited | 2<br>( Day0+21) | IM | 1 |
| **Prophylactic pDNA Vaccine Candidate Against COVID-19** | DNA based vaccine | Imam Abdulrahman Bin Faisal University | 3<br>(Day 0+21+42) | IM | 1 |
| **Booster DNA vaccine delivered by in vivo "EPS Gun" from IGEA optimized for Electro Gene Transfer (EGT) vaccination** | DNA based vaccine | Matti Sällberg, Karolinska Institutet | 1<br>(Day 0) | IM | 1 |
| **SARS-CoV-2 mRNA vaccine (ARCoV)** | RNA based vaccine | AMS, Walvax Biotechnology and Suzhou Abogen Biosciences | 2<br>(Day0+14)<br>Or<br>(Day0+28) | IM | 3 |
| **mRNA-1273.529 - Booster** | RNA based vaccine | ModernaTX, Inc. | 1<br>(Day 0) | IM | 2/3 |
| **CV2CoV, mRNA vaccine** | RNA based vaccine | CureVac AG | 1<br>(Day 0) | IM | 1 |
| **mRNA vaccine (MIPSCo-mRNA-RBD-1)** | RNA based vaccine | University of Melbourne | 1<br>(Day 0) | IM | 1 |
| **A Lyophilized COVID-19 mRNA Vaccine** | RNA based vaccine | Jiangsu Rec-Biotechnology Co., Ltd. | 1<br>(Day 0) | IM | 1 |
| **COVID-19 mRNA Vaccine (SYS6006)** | RNA based vaccine | CSPC ZhongQi Pharmaceutical Technology Co., Ltd. | 2<br>(Day 0+ 21) | IM | 2 |
| **mRNA GEMCOVAC-19 (COVID-19 vaccine)** | RNA based vaccine | Gennova Biopharmaceuticals Limited | 2<br>(Day 0+ 28) | IM | 2/3 |
| **Lyophilized COVID-19 mRNA Vaccine** | RNA based vaccine | Wuhan Recogen Biotechnology Co., Ltd. | 1 | IM | 1 |

| | | | | | |
|---|---|---|---|---|---|
| | | | (Day 0) | | |
| **A self-amplifying RNA (saRNA) boost vaccines (AAHI-SC2 and AAHI-SC3)** | RNA based vaccine | ImmunityBio, Inc. | 1<br>(Day 0) | IM | 1/2 |
| **RQ3013: SARS-CoV-2 mRNA Chimera Vaccine** | RNA based vaccine | Walvax Biotechnology; Shanghai RNACure Biopharma | 1<br>(Day 0) | IM | |
| **mRNA-1273.214 (Booster)** | RNA based vaccine | ModernaTX | 2<br>(Day0+55) | IM | 3 |
| **mRNA-1073; (COVID-19/Influenza) Vaccine** | RNA based vaccine | ModernaTX | 2<br>(Day 0) | IM | 1/2 |
| **RVM-V001** | RNA based vaccine | RVAC Medicines | 1<br>(Day 0) | IM | 1 |
| **ABO1009-DP (COVID-19 Omicron) mRNA Vaccine** | RNA based vaccine | Suzhou Abogen Biosciences Co., Ltd. | 1<br>(Day 0) | IM | 1 |
| **Self-Amplifying Messenger Ribonucleic Acid (samRNA) Vaccines** | RNA based vaccine | Gritstone bio, Inc. | 2<br>(Day0+28) | IM | 1 |
| **Investigational CV0501 mRNA COVID-19 Vaccine** | RNA based vaccine | GlaxoSmithKline | 1<br>(Day 0) | IM | 1 |
| **GLB-COV2-043, an mRNA booster vaccine candidate** | RNA based vaccine | GreenLight Biosciences, Inc. | 1<br>(Day 0) | IM | 1/2 |
| **mRNA-based COVID-19 vaccine (COReNAPCIN)** | RNA based vaccine | ReNAP Technology | 1<br>(Day 0) | IM | 1 |
| **JCXH-221, an mRNA-based** | RNA based vaccine | Immorna Biotherapeutics, Inc. | 1<br>(Day 0) | IM | 1/2 |
| **HDT-301 vaccine** | RNA based vaccine | HDT Bio | 2<br>(Day 0 ± 56) | IM | 1 |
| **EG-COVID vaccine** | RNA based vaccine | EyeGene Inc. | 3<br>(Day 0+21+42) | IM | 1/2 |
| **Coronavirus mRNA vaccine (LVRNA009)** | RNA based vaccine | AIM Vaccine and Liverna Therapeutics | 2<br>(Day0+28) | IM | 3 |
| **VLPCOV-01, self-amplifying RNA vaccine against the coronavirus** | RNA based vaccine | VLP Therapeutics Japan GK | 2<br>NR | IM | 1 |
| **ChulaCov19 mRNA vaccine** | RNA based vaccine | Chulalongkorn University | 2 | IM | 2 |

| | | | | | |
|---|---|---|---|---|---|
| | | | (Day0+21) | | |
| **PTX-COVID19-B, mRNA vaccine** | RNA based vaccine | Providence Therapeutics | 2 (Day0+28) | IM | 3 |
| **CoV2 SAM (LNP) vaccine. A self-amplifying mRNA (SAM) lipid nanoparticle (LNP) platform + Spike antigen** | RNA based vaccine | GlaxoSmithKline | 2 (Day0+30) | IM | 1 |
| **mRNA-1273.351.** | RNA based vaccine | Moderna + National Institute of Allergy and Infectious Diseases (NIAID) | 3 (Day 0 or Day 0 + 28 or Day 56) | IM | 4 |
| **MRT5500, an mRNA vaccine candidate** | RNA based vaccine | Sanofi Pasteur and Translate Bio | 2 (Day0+21) | IM | 2 |
| **DS-5670a,coronavirus-modified uridine RNA vaccine (SARS-CoV-2)** | RNA based vaccine | Daiichi Sankyo Co., Ltd. | 2 NR | IM | 2/3 |
| **HDT-301: Self-replicating mRNA vaccine formulated as a lipid nanoparticle. NA MCTI CIMATEC HDT** | RNA based vaccine | SENAI CIMATEC | 2 (Day0+28) | IM | 2/3 |
| **mRNA-1283** | RNA based vaccine | ModernaTX, Inc. | 2 (Day0+28) | IM | 1 |
| **mRNA COVID-19 vaccine (SW-BIC-213)** | RNA based vaccine | Shanghai East Hospital and Stemirna Therapeutics | 2 TBD | IM | 1 |
| **mRNA-1273.211. A multivalent booster candidate combining mRNA-1273 plus mRNA-1273.351.** | RNA based vaccine | ModernaTX, Inc. | 1 ( Day 0 ) | IM | 2/3 |
| **ARCT-154 mRNA Vaccine** | RNA based vaccine | Arcturus Therapeutics, Inc. | 2 (Day0+28) | IM | 3 |
| **ARCT-165 mRNA Vaccine** | RNA based vaccine | Arcturus Therapeutics, Inc. | 2 (Day0+29) | IM | 1/2 |
| **ARCT-021 mRNA Vaccine** | RNA based vaccine | Arcturus Therapeutics, Inc. | 2 (Day0+30) | IM | 1/2 |
| **VLP vaccines** | | | | | |
| **RBD SARS-CoV-2 HBsAg VLP vaccine** | VLP | Serum Institute of India + Accelagen Pty + SpyBiotech | 2 (Day0+28) | IM | 1/2 |

| | | | | | |
|---|---|---|---|---|---|
| **CoVLP** | VLP | Medicago Inc. | 2 (Day0+21) | IM | 3 |
| **VBI-2902a. An enveloped virus-like particle (eVLP) of SARS-CoV-2 spike (S)** | VLP | VBI Vaccines Inc. | 2 (Day0+28) | IM | 1/2 |
| **SARS-CoV-2 VLP Vaccine** | VLP | The Scientific and Technological Research Council of Turkey | 2 Day 0 | SC | 2 |
| **ABNCoV2 capsid virus-like particle (cVLP) +/- adjuvant MF59** | VLP | Radboud University | 2 (Day0+28) | IM | 3 |
| **SARS-CoV-2 Vaccine LYB001, a receptor-binding domain (RBD) from SARS-CoV-2 and virus-like particle (VLP)** | VLP | Yantai Patronus Biotech Co., Ltd. | 3 (Day0+28+ 56) | IM | 3 |
| **VBI-2901e. The trivalent vaccine composed of virus-like particles (eVLPs) to express the spike proteins of three** | VLP | VBI Vaccines Inc. | 2 (Day0+28) | IM | 1 |
| **Whole-Virus Vaccines** | | | | | |
| **CoronaVac; inactivated SARS-CoV-2 vaccine (vero cell)** | Inactivated virus | Sinovac Research and Development Co., Ltd | 2 (Day0+14) | IM | 4 |
| **Inactivated SARS-CoV-2 vaccine (Vero cell)** | Inactivated virus | Sinopharm | 2 (Day0+21) | IM | 4 |
| **Inactivated SARS-CoV-2 vaccine (Vero cell), vaccine name BBIBP-CorV** | Inactivated virus | Sinopharm | 2 (Day0+21) | IM | 4 |
| **SARS-CoV-2 vaccine (vero cells)** | Inactivated virus | Institute of Medical Biology + Chinese Academy of Medical Sciences | 2 (Day0+28) | IM | 3 |
| **QazCovid-in® - COVID-19 inactivated vaccine** | Inactivated virus | Research Institute for Biological Safety Problems, Rep of Kazakhstan | 2 (Day0+21) | IM | 3 |
| **BBV152 vaccine** | Inactivated virus | Bharat Biotech International Limited | 2 (Day0+14) | IM | 3 |
| **Covi Vax, inactivated coronavirus vaccine** | Inactivated virus | National Research Centre, Egypt | 2 (Day0+28) | IM | 1 |

| | | | | | |
|---|---|---|---|---|---|
| **Inactivated SARS-CoV-2 vaccine (Vero cell)** | Inactivated virus | Shenzhen Kangtai Biological Products Co., Ltd. | 2<br>(Day 0+28) | IM | 3 |
| **COVI-VAC** | Live attenuated virus | Codagenix/Serum Institute of India | 1-2<br>(Day0 or Day0+28) | IN | 3 |
| **VLA2001** | Inactivated Virus | Valneva, National Institute for Health Research, United Kingdom* | 2<br>(Day0+21) | IM | 3 |
| **TURKOVAC, inactivated virus** | Inactivated Virus | Erciyes University and the Health Institutes of Turkey (TUSEB) | 2<br>(Day0+21) | IM | 3 |
| **COVID-19 inactivated vaccine, (CovIran-Barkat)** | Inactivated Virus | Shifa Pharmed Industrial Co | 2<br>(Day0+14) | IM | 2/3 |
| **Inactivated (NDV-based) chimeric vaccine with or without the adjuvant CpG 1018** | Inactivated Virus | The Government Pharmaceutical Organization (GPO); PATH; Dynavax | 2<br>(Day0+28) | IM | 1/2 |
| **Inactivated SARS-CoV-2 vaccine FAKHRAVAC (MIVAC)** | Inactivated Virus | Organization of Defensive Innovation and Research | 2<br>(Day 0 + 14 +/- 21) | IM | 1 |
| **MV-014-212, a live attenuated vaccine that expresses the spike (S) protein of SARS-CoV-2** | Live attenuated virus | Meissa Vaccines, Inc. | 1<br>(Day 0 ) | IN | 1 |
| **Koçak-19 Inactivated adjuvant COVID-19 viral vaccine (TURKOVAC)** | Inactivated Virus | Kocak Farma | 2<br>(Day0+21) | IM | 1 |
| **Adjuvanted inactivated vaccine against SARS-CoV-2** | Inactivated Virus | The Scientific and Technological Research Council of Turkey (TÜBITAK) | 2<br>(Day0+21) | SC | 1 |
| **Inactivated COVID-19 vaccine** | Inactivated Virus | KM Biologics Co., Ltd. | 2<br>(Day0+28) | IM | 3 |

| | | | | | |
|---|---|---|---|---|---|
| **Live recombinant Newcastle Disease Virus (rNDV) vector vaccine** | Inactivated Virus | Laboratorio Avi-Mex | 2<br>(Day0+21) | IM | 2/3 |
| **Inactivated Whole Virion Concentrated Purified Vaccine (CoviVac)** | Inactivated Virus | Chumakov Federal Scientific Center for Research and Development of Immune-and-Biological Products | 2<br>(Day0+14) | IM | 3 |
| **Osvid-19 inactivated vaccine for Covid-19** | Inactivated Virus | Osve Pharmaceutical Company | 2<br>(Day0+28) | IM | 1 |
| **EgyVax Inactivated SARS-CoV-2 vaccine candidate** | Inactivated Virus | Eva Pharma | 2<br>(Day0+14) | IM | 1 |
| **UNAIR Inactivated COVID-19 Vaccine** | Inactivated Virus | Airlangga University, Indonesia | 2<br>(Day0) | IM | 3 |
| **Omicron COVID-19 inactivated Vaccine (Vero Cell)** | Inactivated Virus | China National Biotec Group Company Limited | 2<br>(Day0+28) | IM | 3 |
| **Protein Subunit Vaccine** | | | | | |
| **SARS-CoV-2 rS/Matrix M1-Adjuvant (Full length recombinant SARS CoV-2 glycoprotein nanoparticle vaccine adjuvanted with Matrix M)** | Protein subunit | Novavax | 2<br>(Day0+21) | IM | 3 |
| **Recombinant SARS-CoV-2 vaccine (CHO Cell)** | Protein subunit | Anhui Zhifei Longcom Biopharmaceutical + Institute of Microbiology, Chinese Academy of Sciences | 2-3<br>(Day0+28)<br>Or<br>(Day0+28+56) | IM | 3 |
| **KBP-COVID-19 (RBD-based)** | Protein subunit | Kentucky Bioprocessing Inc. | 2<br>(Day0+21) | IM | 1/2 |
| **VAT00002: SARS-CoV-2 S protein with adjuvant** | Protein subunit | Sanofi Pasteur + GSK | 2<br>(Day0+21) | IM | 3 |

| | | | | | |
|---|---|---|---|---|---|
| **CpG1018/Alum-adjuvanted Recombinant SARS-CoV-2 Trimeric S-protein Subunit Vaccine (SCB-2019)** | Protein subunit | Clover Biopharmaceuticals Inc./Dynavax | 2 (Day0+21) | IM | 3 |
| **COVAX-19® Recombinant spike protein + adjuvant SPIKOGEN®** | Protein subunit | Vaxine Pty Ltd./CinnaGen Co. | 2 (Day0+21) | IM | 3 |
| **MVC-COV1901 (S-2P protein + adjuvant CpG 1018)** | Protein subunit | Medigen Vaccine Biologics + Dynavax + NIAID | 2 (Day0+28) | IM | 4 |
| **FINLAY-FR anti-SARS-CoV-2 Vaccine (RBD + adjuvant)** | Protein subunit | Instituto Finlay de Vacunas * | 2 (Day0+28) | IM | 2 |
| **FINLAY-FR-2 anti-SARS-CoV-2 Vaccine (RBD chemically conjugated to tetanus toxoid plus adjuvant)** | Protein subunit | Instituto Finlay de Vacunas | 2 (Day0+28) | IM | 3 |
| **EpiVacCorona (EpiVacCorona vaccine based on peptide antigens for the prevention of COVID-19)** | Protein subunit | Federal Budgetary Research Institution State Research Center of Virology and Biotechnology "Vector" | 2 (Day0+21) | IM | 3 |
| **RBD (baculovirus production expressed in Sf9 cells) Recombinant SARS-CoV-2 vaccine (Sf9 Cell)** | Protein subunit | West China Hospital + Sichuan University | 2 (Day0+28) | IM | 3 |
| **IMP CoVac-1 (SARS-CoV-2 HLA-DR peptides)** | Protein subunit | University Hospital Tuebingen | 1 (Day0) | SC | 1/2 |

| | | | | | |
|---|---|---|---|---|---|
| **UB-612 (Multitope peptide based S1-RBD-protein based vaccine)** | Protein subunit | Vaxxinity | 2 (Day0+28) | IM | 3 |
| **AdimrSC-2f (recombinant RBD +/- Aluminium)** | Protein subunit | Adimmune Corporation * | NR | NR | 1 |
| **CIGB-669 (RBD+AgnHB)** | Protein subunit | CIGB * | 3 (Day0+14+28) Or (Day0+28+56) | IN | 1/2 |
| **CIGB-66 (RBD+aluminium hydroxide)** | Protein subunit | CIGB * | 3 (Day0+14+28) Or (Day0+28+56) | IM | 3 |
| **BECOV2** | Protein subunit | Biological ELimited | 2 (Day0+28) | IM | 3 |
| **Recombinant Sars-CoV-2 Spike protein, Aluminum adjuvanted (Nanocovax)** | Protein subunit | Nanogen Pharmaceutical Biotechnology* | 2 (Day0+21) | IM | 3 |
| **Recombinant protein vaccine S-268019 (using Baculovirus expression vector system)** | Protein subunit | Shionogi | 2 (Day0+21) | IM | 3 |

| | | | | | |
|---|---|---|---|---|---|
| **SARS-CoV-2-RBD-Fc fusion protein (AKS-452)** | Protein subunit | University Medical Center Groningen + Akston Biosciences Inc. | NR | SC or IM | 2 |
| **COVAC-1 and COVAC-2 sub-unit vaccine (spike protein) + SWE adjuvant** | Protein subunit | University of Saskatchewan | 2 (Day0+28) | IM | 2 |
| **GBP510, a recombinant surface protein vaccine with adjuvant AS03 (aluminium hydroxide)** | Protein subunit | SK Bioscience Co., Ltd. and CEPI | 2 (Day0+28) | IM | 3 |
| **Razi Cov Pars, recombinant spike protein** | Protein subunit | Razi Vaccine and Serum Research Institute | 3 (Day 0 + 21 +51) | IM | 3 |
| **MF59 adjuvanted SARS-CoV-2 Sclamp vaccine** | Protein subunit | The University of Queensland | 2 (Day0+28) | IM | 1 |
| **SK SARS-CoV-2 recombinant surface antigen protein subunit (NBP2001) + adjuvanted with alum.** | Protein subunit | SK Bioscience Co., Ltd. | 2 (Day0+28) | IM | 1 |
| **SpFN (spike ferritin nanoparticle) uses spike proteins with a liposomal formulation QS21 (ALFQ) adjuvant.** | Protein subunit | Walter Reed Army Institute of Research (WRAIR) | 2-3 (Day 0 + 28 + 180) | IM | 1 |
| **EuCorVac-19; A spike protein using the recombinant protein technology and with an adjuvant.** | Protein subunit | POP Biotechnologies and EuBiologics Co.,Ltd | 2 (Day0+21) | IM | 3 |
| **ReCOV: Recombinant two-component spike and RBD protein COVID-19 vaccine (CHO cell).** | Protein subunit | Jiangsu Rec-Biotechnology | 2 (Day0+21) | IM | 3 |

| | | | | | |
|---|---|---|---|---|---|
| **Recombinant SARS-CoV-2 Fusion Protein Vaccine (V-01)** | Protein subunit | Livzon Pharmaceutical | 2 (Day0+21) | IM | 3 |
| **Recombinant SARS-CoV-2 Vaccine (CHO cell)** | Protein subunit | National Vaccine and Serum Institute, China; Beijing Zhong Sheng Heng Yi | 2 (Day 0) | IM | 3 |
| **CoVepiT vaccine: SARS-CoV-2 multi-target peptide vaccine (targeting Spike, M, N, and several non-structural proteins)** | Protein subunit | OSE Immunotherapeutics | 1-2 (Day 0+-21) | SC | 1 |
| **CoV2-OGEN1, protein-based vaccine** | Protein subunit | USSF/Vaxform | 1-2 (Day 0±14) | Oral | 1 |
| **QazCoVac-P - COVID-19 Subunit Vaccine** | Protein subunit | Research Institute for Biological Safety Problems | 1-2 (Day 0+21) | IM | 1/2 |
| **RBD protein recombinant SARS-CoV-2 vaccine (Noora Vaccine)** | Protein subunit | Bagheiat-allah University of Medical Sciences | 3 (Day0+21+35) | IM | 3 |
| **Baiya SARS-CoV-2 VAX1, a plant-based subunit vaccine (RBD-Fc + adjuvant)** | Protein subunit | Baiya Phytopharm Co., Ltd. | 2 (Day 0+21) | IM | 1 |
| **SCB-2020S, an adjuvanted recombinant SARS-CoV-2 trimeric S-protein (from B.1.351 variant)** | Protein subunit | Clover Biopharmaceuticals AUS Pty Ltd | 2 (Day 0+21) | IM | 2 |
| **202-CoV; SARS-CoV-2 spike trimer protein + adjuvant, CpG7909.** | Protein subunit | Shanghai Zerun Biotechnology + Walvax Biotechnology + CEPI | 2 (Day 0+28) | IM | 1/2 |

| | | | | | |
|---|---|---|---|---|---|
| **Recombinant protein RBD fusion dimer adjuvanted vaccine (COVID-19 Vaccine Hipra)** | Protein subunit | Laboratorios Hipra, S.A. | 2<br>(Day 0+21) | IM | 3 |
| **Versamune-CoV-2FC vaccine, recombinant S1 antigen** | Protein subunit | Hospital do Coracao | 3<br>(Day0+28) | NR | 1/2 |
| **SII B.1.351 + Matrix-M1 adjuvant, a monovalent SII SARS-CoV-2 B.1.351 (Beta) variant vaccine** | Protein subunit | Novavax | 2<br>(Day0+21) | IM | 1/2 |
| **SII Bivalent + Matrix-M1 adjuvant, a bivalent SII vaccine containing antigen for both the ancestral strain and B.1.351 (Beta) variant of SARS-CoV-2** | Protein subunit | Novavax | 1<br>(Day0) | IM | 1/2 |
| **SII B.1.617.2 + Matrix-M1 adjuvant, a monovalent SII SARS-CoV-2 B.1.617.2 (Delta) variant vaccine** | Protein subunit | Novavax | 1-2<br>(Day 0 +/-<br>21) | IM | 1/2 |
| **SCTV01C. A Bivalent Recombinant Trimeric S Protein vaccine against SARS-CoV-2 Variants** | Protein subunit | Sinocelltech Ltd. | 1<br>(Day0) | IM | 3 |
| **SARS-CoV-2 Protein Subunit Recombinant Vaccine** | Protein subunit | Bio Farma | 2<br>(Day 0+ 28) | IM | 2/3 |
| **PIKA-Adjuvanted Recombinant SARS-CoV-2 Spike (S) Protein Subunit Vaccine** | Protein subunit | Yisheng Biopharma | 2<br>(Day 0+ 7) | IM | 2/3 |

| | | | | | |
|---|---|---|---|---|---|
| **PepGNP-SARSCoV2, A CD8 T-cell priming adaptive vaccine composed of a Coronaviruses specific peptides mounted on a gold nanoparticle** | Protein subunit | Emergex Vaccines Holding Limited | 2<br>(Day 0+ 21) | ID | 1/2 |
| **SARS-CoV-2 Vaccine (IN-B009)** | Protein subunit | HK inno.N Corporation | 2<br>(Day 0+ 21) | IM | 1 |
| **SARS-CoV-2 Protein Subunit Recombinant Vaccine adjuvanted With Alum+CpG 1018** | Protein subunit | PT Bio Farma | 2<br>(Day 0+ 28) | IM | 3 |
| **Adjuvanted SARS-CoV-2 (COVID-19) Beta Variant RBD Recombinant Protein (DoCo-Pro-RBD-1 + MF59)** | Protein subunit | University of Melbourne | 1<br>(Day 0) | IM | 1 |
| **V-01-351/V-01D Bivalence Vaccine (Omicron) or V-01D-351** | Protein subunit | Livzon Pharmaceutical Group Inc. | 1<br>(Day 0) | IM | 2 |
| **Betuvax-CoV-2 COVID-19 vaccine** | Protein subunit | Human Stem Cell Institute, Russia | 2<br>(Day 0+ 28) | IM | 1/2 |
| **VXS-1223U Microarray patch (HD-MAP) vaccine composed of ARS-CoV-2 spike protein (HexaPro)** | Protein subunit | Vaxxas Pty Ltd | 1<br>(Day 0) | ID | 1 |
| **PRIME-2-CoV_Beta, Orf Virus Expressing SARS-CoV_2 Spike and Nucleocapsid Proteins** | Protein subunit | Speransa Therapeutics | 2<br>(Day 0+ 28) | IM | 1 |

| | | | | | |
|---|---|---|---|---|---|
| **ACM-SARS-CoV-2-beta ACM-CpG vaccine candidate (ACM-001)** | Protein subunit | ACM Biolabs | 2<br>(Day 0+ 28) | IN | 1 |
| **Recombinant spike (rS) (SARS-CoV-2 rS) nanoparticle and quadrivalent hemagglutinin (HA) nanoparticle influenza vaccine (qNIV) combination vaccine with Matrix-M™ adjuvant** | Non covid vaccine+protein subunit | Novavax | 1<br>(Day 0) | IM | 2 |
| **A subunit OMV-linked HexaPro spike vaccine. The vaccine platform is based on outer** | Protein subunit | Intravacc B.V. | 1<br>(Day 0) | IN | 1 |
| **ARVAC-CG vaccine (recombinant protein vaccine against SARS-CoV-2)** | Protein subunit | Laboratorio Pablo Cassara S.R.L. | 2<br>(Day 0+ 28) | IM | 2/3 |
| **Recombinant SARS-CoV-2 S-Trimer Vaccine (CHO Cell) booster** | Protein subunit | Binhui Biopharmaceutical Co., Ltd. | 1<br>(Day 0) | IM | 1 |
| **Subunit recombinant vaccine; Convacell** | Protein subunit | St. Petersburg Research Institute of Vaccines and Sera | 2<br>(Day 0+ 21) | IM | 2/3 |

SARS-COV2, severe acute respiratory syndrome coronavirus; NIAID, National Institute of Allergy and Infectious Diseases; AMS, Academy of Military Science; IN, intranasal; IM, Intramuscular; SC, Subcutaneous; IV, Intravenous; IH, Inhaled; AE, Aerosol; RBD, receptor-binding domain; S, spike; Ad, adenovirus; GM-CSF, Granulocyte-macrophage colony-stimulating factor; ;* Development has been suspended and the candidate vaccine has been removed from the landscape summary analysis. VLP, Virus-Like Particles; CoVLP, Coronavirus-Like Particle COVID-19; VFI, Vaccine Formulation Institute; VIDO, Vaccine and Infectious Disease Organization; CIGB, Center for Genetic Engineering and Biotechnology;

1. W. H. Organization, "COVID-19 vaccine tracker and landscape," Ed., 30 March 2023.
